# Genetic determinants of apixaban plasma levels and their relationship to bleeding and thromboembolic events

**DOI:** 10.3389/fgene.2022.982955

**Published:** 2022-09-14

**Authors:** Sofia Attelind, Pär Hallberg, Mia Wadelius, Anna-Karin Hamberg, Agneta Siegbahn, Christopher B. Granger, Renato D. Lopes, John H. Alexander, Lars Wallentin, Niclas Eriksson

**Affiliations:** ^1^ Department of Medical Sciences, Uppsala University, Uppsala, Sweden; ^2^ Uppsala Clinical Research Center, Uppsala University Hospital, Uppsala, Sweden; ^3^ Duke Clinical Research Institute, Duke Medicine, Durham, NC, United States

**Keywords:** factor Xa inhibitors, apixaban, atrial fibrillation, genome-wide association study, pharmacokinetics, pharmacogenetics, drug-related side effects and adverse reactions

## Abstract

Apixaban is a direct oral anticoagulant, a factor Xa inhibitor, used for the prevention of ischemic stroke in patients with atrial fibrillation. Despite using recommended dosing a few patients might still experience bleeding or lack of efficacy that might be related to inappropriate drug exposure. We conducted a genome-wide association study using data from 1,325 participants in the pivotal phase three trial of apixaban with the aim to identify genetic factors affecting the pharmacokinetics of apixaban. A candidate gene analysis was also performed for pre-specified variants in *ABCB1*, *ABCG2*, *CYP3A4*, *CYP3A5*, and *SULT1A1*, with a subsequent analysis of all available polymorphisms within the candidate genes. Significant findings were further evaluated to assess a potential association with clinical outcome such as bleeding or thromboembolic events. No variant was consistently associated with an altered apixaban exposure on a genome-wide level. The candidate gene analyses showed a statistically significant association with a well-known variant in the drug transporter gene *ABCG2* (c.421G > T, rs2231142). Patients carrying this variant had a higher exposure to apixaban [area under the curve (AUC), beta = 151 (95% CI 59–243), *p* = 0.001]. On average, heterozygotes displayed a 5% increase of AUC and homozygotes a 17% increase of AUC, compared with homozygotes for the wild-type allele. Bleeding or thromboembolic events were not significantly associated with *ABCG2* rs2231142. This large genome-wide study demonstrates that genetic variation in the drug transporter gene *ABCG2* is associated with the pharmacokinetics of apixaban. However, the influence of this finding on drug exposure was small, and further studies are needed to better understand whether it is of relevance for ischemic and bleeding events.

## 1 Introduction

Since their introduction into clinical routine, direct oral anticoagulants (DOACs) that inhibit thrombin (dabigatran) or factor Xa (rivaroxaban, apixaban, and edoxaban) have largely replaced warfarin as the first-hand choice to protect against ischemic stroke in patients with atrial fibrillation. Although safer than warfarin, the risk of major bleeding during treatment with a DOAC is estimated to 2%–3% per year (Connolly et al., 2009; Granger et al., 2011; Patel et al., 2011; Giugliano et al., 2013); the risk of intracranial haemorrhage is estimated to about 0.1%–0.5% per year, and the risk of major gastrointestinal bleeding to 1%–1.5%. Lack of efficacy to anticoagulant therapy also has potentially severe consequences. In clinical trials of atrial fibrillation, DOAC treatment was associated with a yearly incidence of ischemic stroke of about 1%–1.5% and of systemic embolism of about 0.05%–0.15% (Connolly et al., 2009; Granger et al., 2011; Patel et al., 2011; Giugliano et al., 2013).

Among factors known to affect the risk of complications to DOAC treatment are those that influence exposure to the drug, such as dose, renal function, and concomitant treatment with interacting drugs. Genetic variation influencing drug levels may also affect this risk. Using data from the RE-LY trial, a genome-wide association study (GWAS) showed that genetic variation in *CES1*, which encodes the liver enzyme carboxylesterase 1 that activates the prodrug dabigatran etexilate, is associated with lower concentrations of dabigatran, and lower risk of bleeding (Pare et al., 2013).

Apixaban is eliminated in several ways of which renal excretion amounts to around 26% (Raghavan et al., 2009). Metabolic pathways include O-demethylation and hydroxylation mainly through CYP3A4 (Asic et al., 2018). SULT1A1 plays a major role for the formation of the first inactive metabolite O-demethyl apixaban sulphate (Nagar et al., 2006; Raghavan et al., 2009; Wang et al., 2009; Asic et al., 2018; Kanuri and Kreutz, 2019; Pharmacogenomics and Pharmacogenetics, 2022). Apixaban is also a substrate of the efflux transporters BCRP encoded by *ABCG2* and P-gp encoded by *ABCB1* (Eliquis, 2022). According to two small candidate gene studies, single nucleotide polymorphisms (SNPs) in *CYP3A5* and *ABCG2* were associated with higher concentration of apixaban (Ueshima et al., 2017; Ueshima et al., 2018). In a candidate gene study on 358 patients, *ABCG2* genotype contributed to interpatient apixaban variability beyond known clinical factors, while genotyped variants of *ABCB1*, *CYP3A4*, and *CYP3A5* had no effect (Gulilat et al., 2020). However, an intronic *ABCB1* variant has been associated with lower apixaban peak concentrations (Dimatteo et al., 2016) and with a lower risk of bleeding events in apixaban users (Lahteenmaki et al., 2021) in small studies. In another candidate gene study on 164 patients, known polymorphisms of *CYP3A4* and *ABCB1* did not affect the exposure to apixaban measured as the area under the curve (AUC) (Lenoir et al., 2022).

To our knowledge, no GWAS on the PK of apixaban has been published (GWAS Catalog, 2022).

## 2 Objective

In this study, we used genome-wide data from a phase three trial of apixaban with the aim to identify genetic factors potentially affecting the pharmacokinetics of apixaban. Significant findings were further evaluated to assess the association with clinical outcome such as bleeding or thromboembolic events.

## 3 Materials and methods

We analysed data from patients who participated in a large phase three clinical trial for apixaban (ARISTOTLE). The ARISTOTLE trial was completed in 2011 and included in the marketing authorisation application for ELIQUIS (apixaban) (Granger et al., 2011). Patients in this trial (*n* = 18,201) were adult individuals with atrial fibrillation randomised to treatment with apixaban or warfarin and followed for a median of 1.8 years. Data from whole-genome scans and clinical outcome (major bleeding, clinically relevant non-major bleeding, and stroke or systemic embolism) were available for 2,800 apixaban-treated patients, of which 1,325 also had PK data (Table 1 and Supplementary Table S1).

**TABLE 1:** Characteristics for apixaban-treated patients with data from both whole-genome scans and pharmacokinetic evaluations.

	N	a/b/c	Mean ± SD
Age	1,325	63/70/76	69 ± 9
Patient sex: Female	1,325	33% (438)	
Height (cm)	1,322	163.5/171.0/178.0	170.8 ± 10.4
Weight (kg)	1,323	74/86/99	88 ± 20
BMI (kg/m^2^)	1,322	26.1/29.3/33.1	30.0 ± 5.8
Smoker	1,325	9% (120)	
**Race**	1,325		
White		94% (1243)	
Black/African American		1% (8)	
Asian		5% (71)	
Other		0% (3)	
**Region**	1,325		
Asia/Pacific		7% (96)	
Europe		53% (705)	
Latin America		5% (65)	
North America		35% (459)	
Serum creatinine at baseline (mg/dl)	1,325	0.88/1.02/1.20	1.07 ± 0.28
Calculated CrCl at baseline (ml/min)	1,323	59.2/75.9/99.2	82.2 ± 32.9
GFR CKD-EPI (ml/min/1.73 m^2^)	1,325	55.8/68.5/81.6	68.2 ± 17.3
**CHADS-2 score**	1,325		
0		1% (7)	
1		34% (447)	
2		36% (476)	
3		19% (250)	
4		8% (108)	
5		2% (33)	
6		0% (4)	
Prior stroke/TIA/SEE	1,325	18% (244)	
Prior stroke	1,325	10% (134)	
Diabetes	1,325	26% (340)	
Hypertension (treated)	1,325	88% (1164)	
Myocardial infarction	1,325	14% (186)	
Heart failure	1,325	28% (367)	
CHF within 3 months or LVEF ≤40%	1,325	32% (428)	
History of clinically relevant or spontaneous bleeding	1,325	19% (252)	
ACEi/ARB	1,325	72% (960)	
Amiodarone	1,325	8% (108)	
Beta blocker	1,325	66% (878)	
Aspirin	1,325	28% (366)	
Clopidogrel	1,325	1% (13)	
Digoxin	1,325	34% (444)	
Calcium channel blocker	1,325	32% (423)	
Statin	1,325	45% (596)	
Nonsteroidal anti-inflammatory agent	1,325	11% (143)	
Dose: 5 mg (twice daily)	1,325	96% (1266)	
**PK-parameters**			
AUC_ss_	1,325	2832.9/3501.5/4220.3 (494.6, 8600.9)	
C_max,ss_	1,325	128.9/163.0/204.7 (31.3, 477.9)	
C_min,ss_	1,325	74.7/102.5/137.5 (4.00, 376.8)	
**Clinical outcomes** ^a^			
Major bleeding	2,799	4% (105)	
Major or CRNM bleeding	2,799	8% (214)	
Haemorrhagic stroke	2,800	0.4% (11)	
Ischemic stroke	2,800	1% (32)	

a/b/c represents the lower quartile a, the median b, and the upper quartile c for continuous variables. Numbers after percentage are frequencies. For PK-parameters (x, y) represents x = min and y = max. N is the number of non-missing values. SD, standard deviation; BMI, body mass index; CrCl, creatinine clearance (Cockcroft-Gault); GFR, glomerular filtration rate; CKD-EPI, chronic kidney disease epidemiology collaboration; TIA, transitory ischemic attack; SEE, systemic embolic event; CHF, congestive heart failure; LVEF, left ventricular ejection fraction; ACEi, angiotensin converting enzyme inhibitor; ARB, angiotension II receptor blocker; AUC_ss_, area under the curve at steady state; C_max ss_, maximum serum concentration at steady state; C_min ss_, serum trough concentration at steady state; CRNM, clinically relevant non-major.

a Includes patients with data from whole-genome scans, with or without pharmacokinetic data.

The phase three clinical trial (ARISTOTLE study ID: NCT00412984) was approved by the appropriate ethics committees at all study sites. All patients provided written informed consent before enrolment.

Genetic associations with the PK of apixaban were investigated by utilising data on the PK parameters area under the plasma concentration time curve at steady state (AUC_ss_), the maximum concentration at steady state (C_max,ss_) as well as the trough concentration at steady state (C_min,ss_) (Cirincione et al., 2018). Any significant findings were tested for association with bleeding or thromboembolic events by analysing the primary and secondary outcomes as specified in the ARISTOTLE trial (Granger et al., 2011).

We performed an association study covering the entire genome and pre-specified variants in candidate genes that encode transport proteins and metabolic enzymes involved in the PK of apixaban. These candidate variants have previously been suggested to influence blood concentrations of apixaban or outcome of the anticoagulant treatment (Table 2). We further did an analysis of all available polymorphisms within these candidate genes and included a region covering ±10 kb upstream and downstream of each gene (Supplementary Table 2).

**TABLE 2:** List of candidate single nucleotide polymorphisms.

Gene and variant	SNP	Chromosome	Position, base pair	Minor allele	Major allele	MAF	Imputation quality	Genotyped 1 or imputed 0
ABCB1 c.3435 (Ueshima et al., 2018; Lahteenmaki et al., 2021)	rs1045642	7	87138645	G	A	0.4672	1.00	1
ABCB1 c.2677 (Ueshima et al., 2018; Lahteenmaki et al., 2021)	rs2032582A/C	7	87160618	A	C	0.4535	0.98	0
ABCB1 c.2677 (Ueshima et al., 2018; Lahteenmaki et al., 2021)	rs2032582T/A	7	87160618	T	A	0.0385	0.92	0
ABCB1 c.1236 (Ueshima et al., 2018; Lahteenmaki et al., 2021)	rs1128503	7	87179601	A	G	0.4604	1.00	1
ABCG2 c.421 (Ueshima et al., 2017; Ueshima et al., 2018)	rs2231142	4	89052323	T	G	0.1177	1.00	1
ABCG2 c.34 (Gulilat et al., 2020)	rs2231137	4	89061114	T	C	0.0679	0.98	0
CYP3A5 *3 (Ueshima et al., 2017; Ueshima et al., 2018)	rs776746	7	99270539	T	C	0.0936	1.00	1
CYP3A4 *22 (Lenoir et al., 2022)	rs35599367	7	99366316	A	G	0.0366	1.00	1
CYP3A4 *1B (Lenoir et al., 2022)	rs2740574	7	99382096	C	T	0.0468	0.98	0
SULT1A1 *2 (Kanuri and Kreutz, 2019)	rs1042028	16	28617514	T	C	0.3192	0.93	0

SNP, single nucleotide polymorphism; MAF, minor allele frequency.

### 3.1 Genome-wide array data and analyses

DNA was extracted from whole blood using NucleoSpin^®^ 96 Blood kit (from MACHEREY-NAGEL). Genotyping was performed on the Illumina Global Screening Array 24v2 at the SNP&SEQ Platform of the Science for Life Laboratory at Uppsala University. Genotype calls were generated using Illumina GenomeStudio 2.0.3.

Quality control (QC) of the raw genotyped data included exclusion of patient sex mismatches, related patients with PLINK pi_hat >0.2, variants with more than 2% missing information, variants with Hardy-Weinberg Equilibrium *p*-value < 5 × 10^−8^, and variants with Minor Allele Frequency (MAF) < 0.1%. The pre-imputation number of variants was 541,772 and the number of patients was 5,553 individuals of which 2,800 were treated with apixaban and included in this study. Data on clinical outcome was available for all 2,800 individuals and PK data was available for 1,325 individuals.

Pre-imputation QC was performed versus the Haplotype Reference Consortium (HRC) panel using McCarty Group Tools QC Script (v 4.2.9) (Scattershot, 2021). Imputation was performed with Positional Burrows-Wheeler transform (PBWT) (Durbin, 2014) using the HRC r1 reference panel (McCarthy et al., 2016) with the Sanger imputation server. The program used for phasing was Eagle v2.3.3 (Loh et al., 2016).

Post-imputation, variants with MAF < 0.1% were excluded and the data was filtered on imputation quality above 0.7 and converted to hard calls using PLINK v1.9. The total number of SNPs post-imputation and QC was 16.7 million. In our GWAS analysis, we reported variants with MAF >1% (7.9 million).

To account for possible population stratification, principal component analysis (PCA) was performed using PLINK v1.9. The first six genetic principal components were included in all analyses. Comparison between the ARISTOTLE genetic population and HapMap was performed by performing PCA on the merged ARISTOTLE and HapMap release 23 material (Supplementary Figure 1). For the GWAS analyses, separate sensitivity analyses were performed in the largest cluster based on K-means clustering of genetic PCA (Statistical methods, Supplementary Figure 2).

### 3.2 Statistical methods

The method presented by Takeuchi et al. (2009) was used to estimate the power of the GWAS analysis for a marker explaining specific amounts of variance (R^2^). For the clinical analyses, we estimated the power using the GPC function in the R-package *Genetics Design* for the outcomes major bleeding and major/clinically relevant non-major bleeding (Supplementary Figures 3–5).

All analyses including genetic variants assumed an additive genetic model, i.e., SNPs were coded 0, 1, and 2 according to the number of minor alleles. GWAS, candidate SNP, and gene analyses were performed using linear regression as implemented in PLINK v1.9. The analyses were adjusted for age, patient sex, calculated creatinine clearance (CrCl) at baseline (ml/min), weight (kg), dose of apixaban, concomitant treatment with amiodarone or calcium channel blockers and the first six genetic principal components. These variables were selected as they are known to affect the PK of apixaban (Cirincione et al., 2018). Chromosome X was analysed assuming no chromosome X inactivation as implemented in PLINK. Possible inflation in *p*-values was measured by genomic inflation factor lambda and by visually inspecting QQ plots.

In the PK analyses, two missing values on weight were imputed and in the clinical outcome analyses, five missing values of weight and four of calculated CrCl at baseline were imputed. The imputation was performed using the R-package mice with the default settings.

The sensitivity population was defined by performing K-means clustering on the first two genetic principal components. The number of clusters was determined by the elbow method, i.e., the number of clusters was set at the point where there was little change in within-cluster variance per increase of one cluster. Our optimal number of clusters was 4 and the largest cluster was used as the sensitivity population.

The coefficient of determination (R^2^) was used to describe the amount of variation explained by a regression model (including multiple and univariate models). The partial R^2^, here defined as the increase in R^2^ of a model when a variable is added last, was used to quantify the impact of a single variable on a multiple linear regression model.

The significance level for the candidate SNP and gene analyses was set to the Bonferroni adjusted limit of 0.05/number of tests. The level of GWAS significance was set to the conventional threshold of *p* < 5 × 10^−8^ (Sham and Purcell, 2014).

Time to event was analysed using Cox proportional hazard regression analyses adjusted by the same covariates as in the GWAS. The threshold for significance in the Cox regression analyses was set at the Bonferroni cut-off according to 0.05/number of tests. The 95% confidence intervals for the incidence rates were estimated using a gamma distribution.

Analyses other than candidate SNP, gene, and GWAS were performed using R version 3.6.0 (R Foundation for Statistical Computing, Vienna, Austria).

### 3.3 Clinical outcomes

#### 3.3.1 Pharmacokinetic data

Measures of apixaban exposure used in the analyses included AUC_ss_, C_max,ss_, and C_min,ss_ and were received from the ARISTOTLE study team (Granger et al., 2011). Individual exposures were derived from empirical Bayes estimates of individual PK parameters using sparse data (single sample at month two in the ARISTOTLE study) and a previously developed population PK model described by Cirincione et al. (2018).

#### 3.3.2 Bleeding and thromboembolic events

Bleeding and thromboembolic events were defined according to the criteria specified in the ARISTOTLE trial (Granger et al., 2011).

Major bleeding as defined by the International Society on Thrombosis and Haemostasis (ISTH) were acute or sub-acute clinically overt bleeding accompanied by a decrease in haemoglobin level of ≥2 g/dl, and/or a transfusion of ≥2 U of packed red blood cells, and/or bleeding that was fatal or occurred in the following critical sites: intracranial, intra-spinal, intra-ocular, pericardial, intra-articular, intra-muscular with compartment syndrome, or retroperitoneal (Granger et al., 2011).

Clinically relevant non-major bleeding (CRNM) was defined as acute or sub-acute clinically overt bleeding that did not satisfy the criteria for major bleeding and led to hospital admission for bleeding, physician-guided medical or surgical treatment for bleeding, or a change in antithrombotic therapy (including study drug) for bleeding. All acute clinically overt bleeding events not meeting criteria for major bleeding or clinically relevant non-major bleeding were classified as minor bleeding (Granger et al., 2011).

Stroke was defined as a non-traumatic focal neurologic deficit lasting ≥24 h, including retinal ischemic events (embolism or thrombosis). Strokes were classified as ischemic, ischemic with haemorrhagic transformation, haemorrhagic, or stroke of uncertain type. Systemic embolism required a clinical history consistent with an acute loss of blood flow to a peripheral artery supported by evidence of embolism from surgical specimens, autopsy, angiography, vascular imaging, or other objective testing (Granger et al., 2011).

## 4 Results

Baseline characteristics of the PK population are presented in Table 1. The median age of 70 years and patient sex distribution with 33% females were similar to the main ARISTOTLE trial population. The self-reported ethnicity was predominantly white (94%), and patients were mainly included from Europe (53%), and North America (35%). A total of 1,266 patients received the standard dose of 5 mg twice daily, and 59 the reduced dose of 2.5 mg twice daily. The reduced dose is recommended in patients fulfilling at least two of the following criteria: age ≥ 80 years, body weight ≤ 60 kg, and serum creatinine ≥ 1.5 mg/dl (133 µmol/L) (Eliquis, 2022).

Linear regression results for the covariates used in the genetic models versus the apixaban exposure variable AUC_ss_ are presented in Table 3. The overall model R^2^ was 0.45. The three variables with highest partial R^2^ were dose of apixaban followed by calculated CrCl at baseline (ml/min) and patient sex.

**TABLE 3:** Area under the curve at steady-state (AUC_ss_)—Linear regression results for covariates used in the analyses. N = 1,323^a^.

Variable	Comparison	Beta	CI	P	R^2^	Variable R^2^	Partial R^2^
Age	By increase of one SD: 9.14	128.012	(69.627–186.396)	1.86 × 10^−5^	0.458	0.106	0.008
Patient sex	Female vs. Male	631.157	(535.03–727.284)	8.78 × 10^−36^	0.458	0.113	0.068
Calculated CrCl at baseline (ml/min)	By increase of one SD: 32.8	−559.071	(−636.089–482.054)	7.82 × 10^−43^	0.458	0.235	0.083
Weight (kg)	By increase of one SD: 20.36	75.281	(9.557–141.006)	2.49 × 10^−2^	0.458	0.112	0.002
Dose	5 vs. 2.5	2008.575	(1786.599–2230.552)	2.88 × 10^−63^	0.458	0.037	0.13
Amiodarone	Yes vs. No	433.036	(274.07–592.002)	1.10 × 10^−7^	0.458	0.009	0.012
Calcium channel blocker	Yes vs. No	276.504	(184.124–368.885)	5.63 × 10^−9^	0.458	0.012	0.014

R^2^, amount of variance explained by the model; Variable R^2^, amount of variance explained by the variable on its own; Partial R^2^, the increase in amount of variance explained if the variable is added last to a model including all other variables; SD, standard deviation; CrCL, creatinine clearance (Cockcroft-Gault).

a Weight was subsequently imputed in two patients, and genetic associations with AUC were calculated on all 1,325 patients.

### 4.1 Genome-wide association study analyses

With 1,325 observations we had the power to detect a variant explaining approximately 1.5% of the variance (R^2^) in the PK outcomes (Supplementary Figure 3).

**FIGURE 1:**
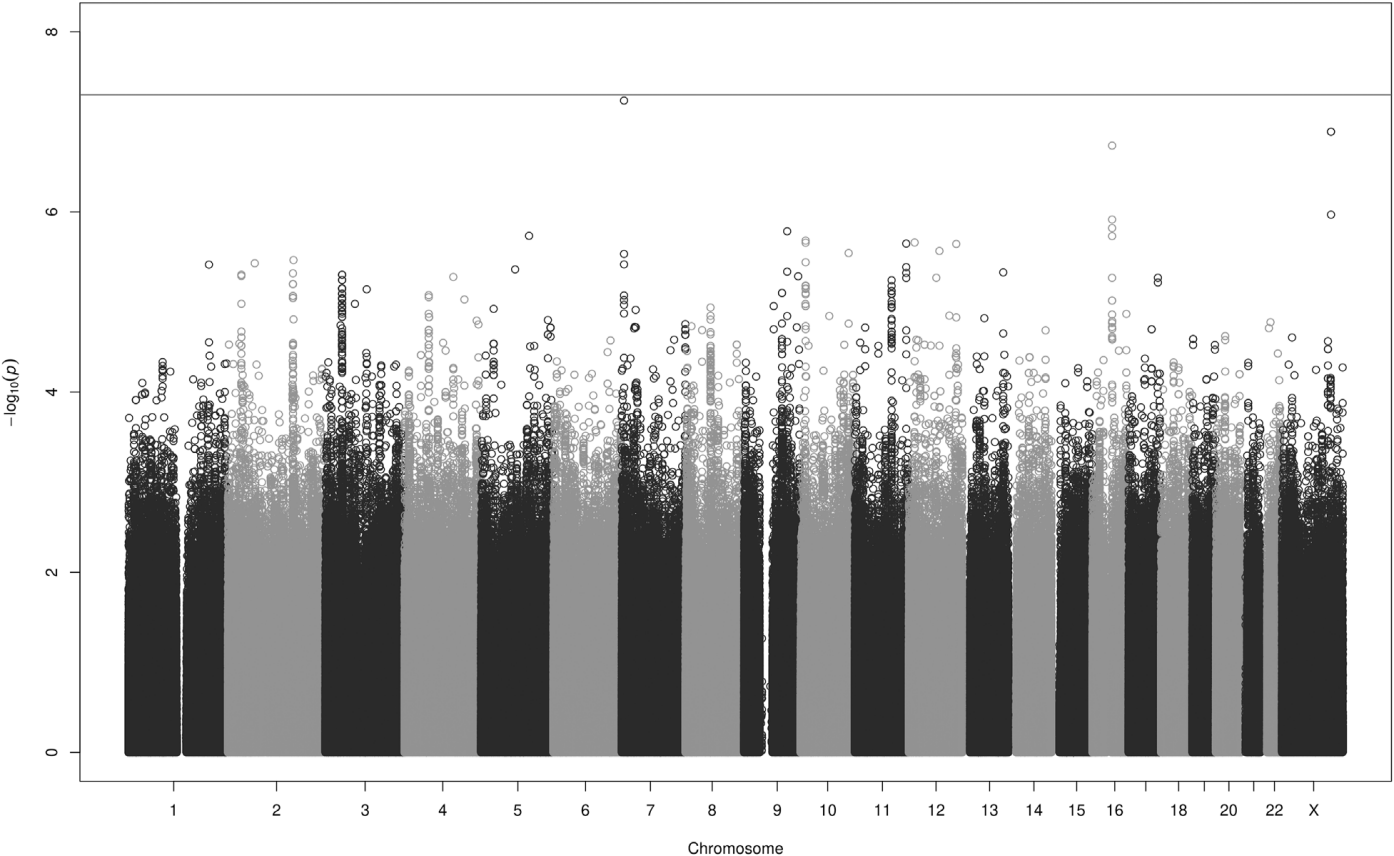
Manhattan plot for the GWAS analyses of apixaban AUC_ss_ in 1,325 patients adjusted for the covariates listed in Table 3. The grey line denotes the genome-wide significance level *p* < 5 × 10^−8^.

Results of the GWAS analyses of AUC_ss_, C_max,ss_, and C_min,ss_ in 1,325 patients are shown in the Manhattan plots in Figure 1 (AUC_ss_) and in Supplementary Figures 6, 7 and Supplementary Tables 3–5. No inflation in *p*-values was observed with lambda values of 1.006, 1.006, and 1.007 for the analyses of AUC_ss_, C_max,ss_, and C_min,ss_, respectively.

The Manhattan plot for C_min,ss_ (Supplementary Figure 7) indicated a signal in an intergenic region on chromosome X. This rare SNP (rs147256925), which is not in linkage disequilibrium (LD, r^2^ ≥ 0.2) with any nearby SNP (Ward and Kellis, 2012) and has a MAF just above the threshold for QC, is most likely an artefact. Its closest neighbouring gene, gastrin releasing peptide receptor (*GRPR*) is located about 100k bases away. Gastrin-Releasing Peptide (GRP) regulates functions of the gastrointestinal and central nervous system, such as release of gastrointestinal hormones, smooth muscle contraction, and epithelial cell proliferation. The SNP rs147256925 has no evidence of a regulatory function (National Center for Biotechnology Information NIH National, 2022). Except for this SNP, no single variant reached genome-wide significance.

There were, however, several nominally significant associations with *p*-values <1 × 10^−6^. For the outcome AUC_ss_ these included rs111844911 on chromosome 7, rs183109587 and rs184794076 on chromosome X, and rs59884489 on chromosome 16. For the outcome C_min,ss_ they included rs78896694 on chromosome 7, rs143178045 on chromosome 8, and rs12107681 on chromosome 3, and for the outcome C_max,ss_, rs56293342 on chromosome 2. A description of these SNPs and their closest genes can be found in the Supplementary Material. These SNPs and genes were not connected to an obvious or plausible biological mechanism impacting the PK of apixaban and have, to our knowledge, not previously been associated with altered drug PK. It is likely that they are spurious results.

Sensitivity GWAS analyses in 1,174 patients of mainly white ethnicity according to the PCA gave the same results as the main analyses (Supplementary Figures 8–10).

### 4.2 Candidate gene analyses of pre-specified variants

**FIGURE 2:**
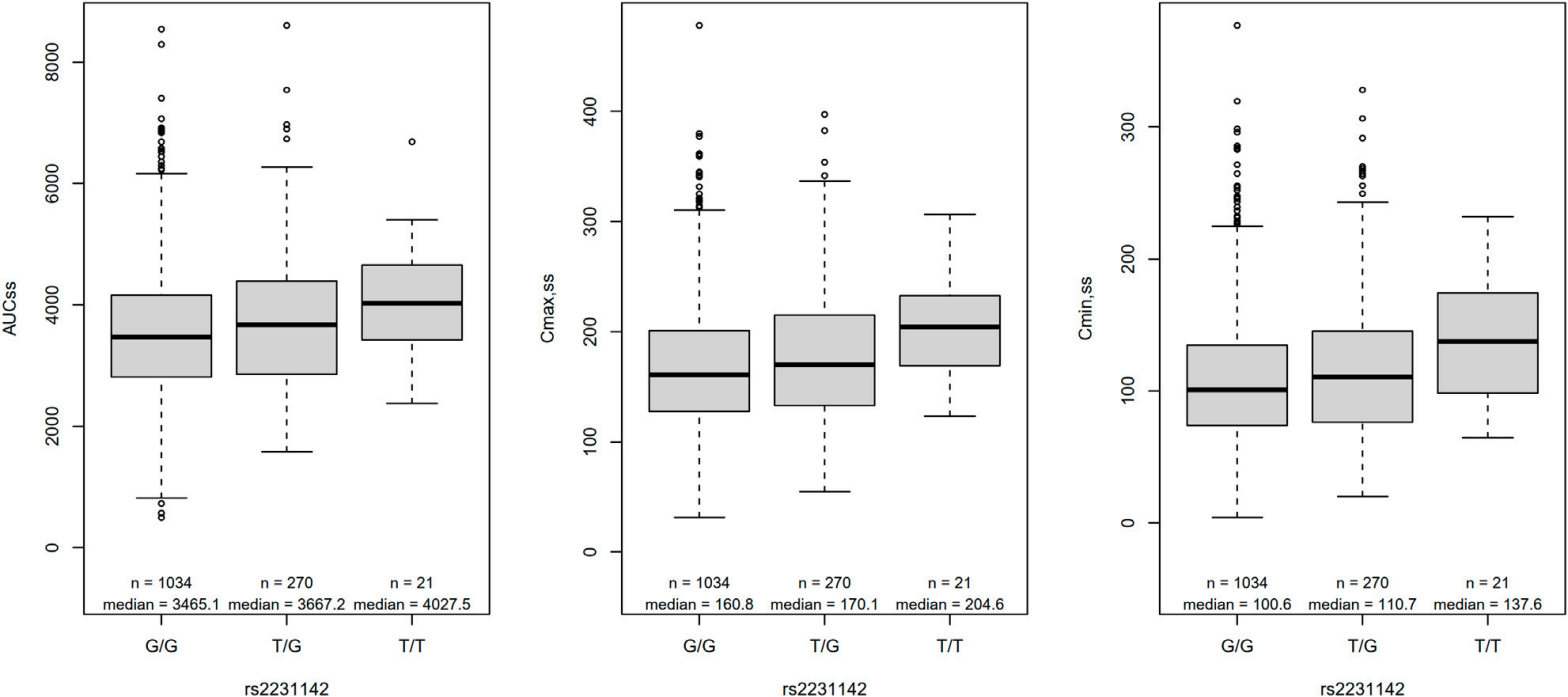
Box-plot of the PK parameters by the SNP rs2231142. AUC_ss_, area under the curve at steady state; C_max,ss_, maximum serum concentration at steady state; C_min,ss_, serum trough concentration at steady state; n, number of genotype carriers; the box represents the lower and upper quartile whereas the thick middle line denotes the median. The whiskers extend to the maximum and minimum value within 1.5 times the inter quartile range and values outside of these limits are presented as circles.

**FIGURE 3:**
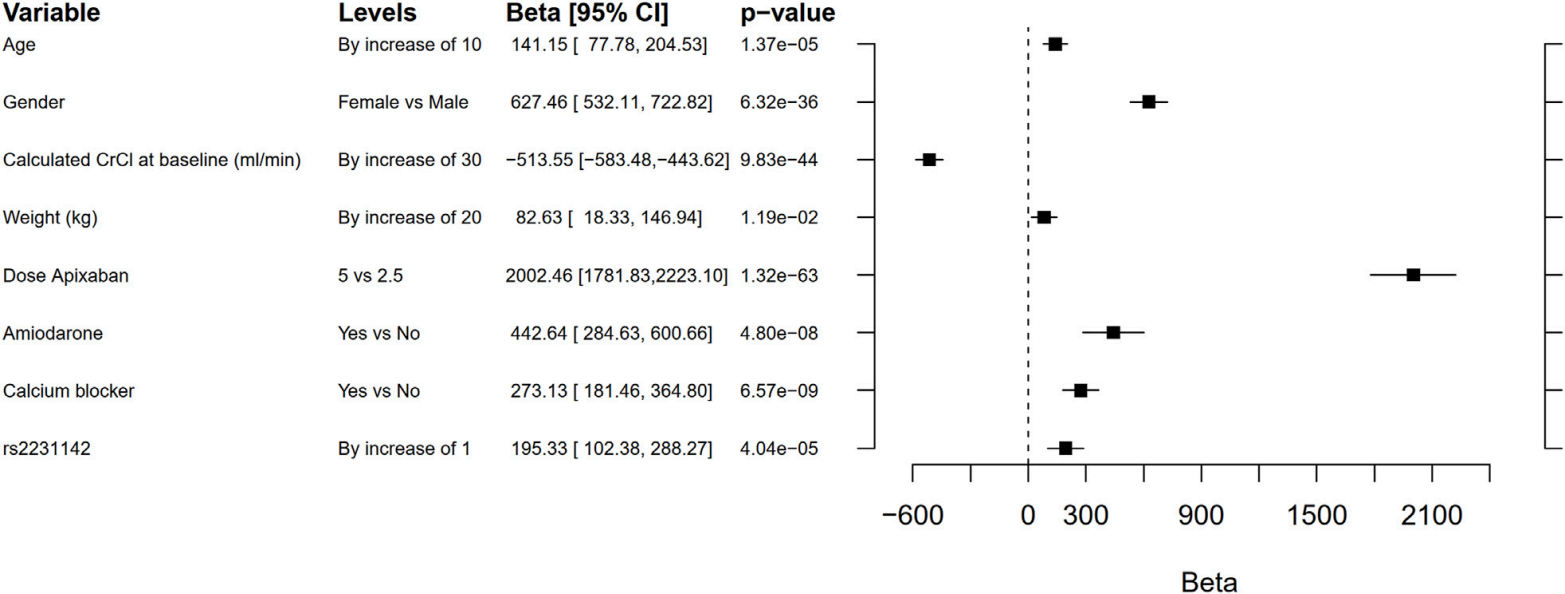
Forest plot comparing the effect on area under the curve (AUC) of rs2231142 with that of age, gender, creatinine clearance (CrCL), weight, dose, co-medication with amiodarone or calcium blockers. The beta coefficient is the degree of change in the outcome variable for every unit of change in the predictor variable. Note that these results are not adjusted by genetic principal components 1 to 6. The Bonferroni threshold for significance was *p* < 0.0045.

The candidate gene analyses of the pre-specified variants in *ABCB1*, *ABCG2*, *CYP3A4*, *CYP3A5*, and *SULT1A1* (Table 2 and Supplementary Table 2) in relation to AUC_ss_, C_max,ss_, and C_min,ss_ are shown in Table 4. The number of candidate SNPs was 11, hence the Bonferroni threshold for significance was *p* < 0.0045. The SNP rs2231142 in *ABCG2* on chromosome 4 was statistically significantly associated with all three PK outcomes. Descriptive statistics of the PK parameters by the SNP rs2231142 are shown in Figure 2. Linear regression results for the SNP rs2231142 are shown in Figure 3 and Supplementary Table 6. The effect was small and explained about 1% of the total variance. On average, heterozygotes displayed a 5% increase of AUC_ss_ and homozygotes 17%, compared with homozygotes for the wild-type allele. We did not find any association between other candidate SNPs and the PK outcomes.

**TABLE 4:** Association between candidate single nucleotide polymorphisms and AUC_ss_, C_max,ss_, and C_min,ss_. N = 1,325.

Gene	SNP	Alleles minor/major	Variable	Beta	Confidence interval lower 95	Confidence interval upper 95	*p*
ABCB1	rs1045642	G/A	AUC_ss_	31.66	−28.71	92.03	0.3042
			C_max,ss_	2.33	−1.417	6.077	0.2231
			C_min,ss_	1.714	−1.665	5.092	0.3203
ABCB1	rs2032582A/C	A/C	AUC_ss_	−7.011	−65.64	51.61	0.8147
			C_max,ss_	−0.8717	−4.511	2.767	0.6388
			C_min,ss_	−0.9038	−4.184	2.377	0.5893
ABCB1	rs2032582T/A	T/A	AUC_ss_	−61.480	−217.90	94.97	0.4413
			C_max,ss_	−3.2330	−12.950	6.481	0.5143
			C_min,ss_	−3.7170	−12.470	5.038	0.4055
ABCB1	rs4148738	C/T	AUC_ss_	−1.429	−60.31	57.45	0.9621
			C_max,ss_	−0.8235	−4.478	2.831	0.6588
			C_min,ss_	−0.7336	−4.028	2.561	0.6626
ABCB1	rs1128503	A/G	AUC_ss_	−1.962	−61.42	57.50	0.9484
			C_max,ss_	−0.8854	−4.576	2.806	0.6383
			C_min,ss_	−1.031	−4.358	2.296	0.5436
ABCG2	rs2231142	T/G	AUC_ss_	151	59.15	242.8	0.0013*
			C_max,ss_	9.633	3.934	15.33	0.0009*
			C_min,ss_	9.695	4.562	14.83	0.0002*
ABCG2	rs2231137	T/C	AUC_ss_	−68.3	−187.3	50.73	0.2610
			C_max,ss_	−4.973	−12.36	2.414	0.1873
			C_min,ss_	−4.652	−11.31	2.007	0.1712
CYP3A5	rs776746	T/C	AUC_ss_	−68.72	−175.1	37.64	0.2056
			C_max,ss_	−5.425	−12.03	1.175	0.1074
			C_min,ss_	−4.493	−10.44	1.457	0.1391
CYP3A4	rs35599367	A/G	AUC_ss_	42.74	−113.6	199.1	0.5921
			C_max,ss_	0.2975	−9.408	10.00	0.9521
			C_min,ss_	−0.6454	−9.394	8.103	0.8851
CYP3A4	rs2740574	C/T	AUC_ss_	−61.18	−205.3	82.94	0.4055
			C_max,ss_	−5.929	−14.87	3.014	0.1940
			C_min,ss_	−4.216	−12.28	3.848	0.3057
SULT1A1	rs1042028	T/C	AUC_ss_	17.830	−46.52	82.17	0.5872
			C_max,ss_	0.7968	−3.198	4.792	0.6959
			C_min,ss_	1.375	−2.225	4.976	0.4542

*p-values lower than the Bonferroni threshold of significance of 0.0045. AUC_ss_, area under the curve at steady state; C_max ss_, maximum serum concentration at steady state; C_min ss_, serum trough concentration at steady state. Note that these results are adjusted by genetic principal components 1 to 6.

### 4.3 Candidate gene analyses of all available variants

The total number of SNPs available for analysis in a region covering ±10 kb upstream and downstream of each gene *ABCB1*, *ABCG2*, *CYP3A4*, *CYP3A5*, and *SULT1A1* after QC was 866, and the threshold for significance was therefore set to 5.77 × 10^−5^. No variant was statistically significantly associated with AUC_ss_, C_max,ss_, or C_min,ss_ (Supplementary Table 7 shows the top ten SNPs per PK parameter and Supplementary Tables 8–10 the top 60 variants).

### 4.4 Bleeding and thromboembolic events

With 2,799 observations, we had the power to detect a variant with a hazard ratio slightly below 2 for the outcome major bleeding and around 1.5 for the outcome major/clinically relevant non-major bleeding (Supplementary Figures 4, 5).

Clinical outcomes are shown in Table 1 and Supplementary Table 1. One candidate variant (rs2231142) passed the Bonferroni threshold for significance and was moved forward to analyses versus clinical events.

**FIGURE 4:**
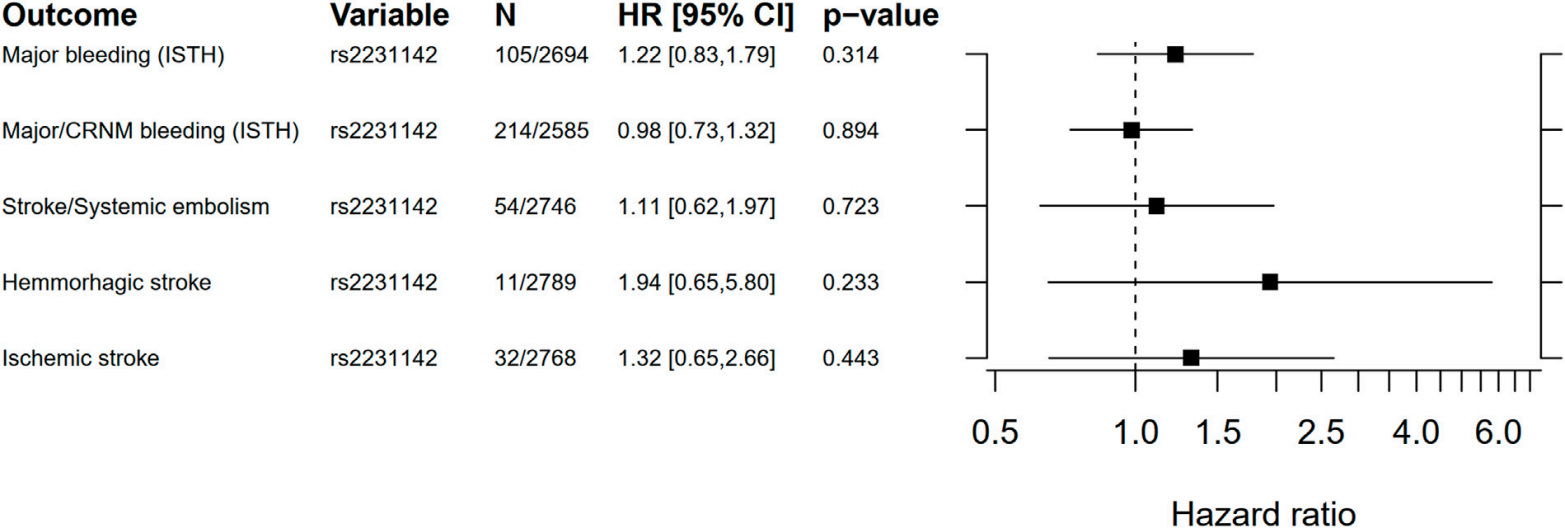
Forest plot for the Cox proportional hazard ratio for carrying rs2231142 versus bleeding and thromboembolic events. Ninety five percent confidence intervals (CI) for incidence rates were estimated using a gamma distribution. ISTH, International Society on Thrombosis and Haemostasis; CRNM, clinically relevant non-major bleeding.

Cox proportional hazard regression analyses results for the candidate SNP rs2231142 versus bleeding and thromboembolic events are shown in Figure 4, and incidence rates per genotype are presented in Supplementary Table 11. No statistically significant associations were found.

## 5 Discussion

In this genome-wide study, no genetic variant was consistently associated with the PK of apixaban on a genome-wide level. In a pre-specified candidate SNP analysis, rs2231142 in the drug transporter gene *ABCG2* (Heyes et al., 2018) was significantly associated with the PK parameters AUC_ss_, C_max,ss_, and C_min,ss_. This variant led to a higher exposure to apixaban and could explain about 1% of the variance [AUC_ss_, beta = 151 (95% CI 59–243), *p* = 0.001]. Compared with homozygotes for the wild-type allele, the AUC_ss_ increased on average 5% in heterozygotes and 17% in homozygotes. This replicates the finding in previous studies by Ueshima et al. (2017, 2018), although the magnitude of the effect was lower in our study. For comparison, patient sex explains notably more of the variance in AUCss [beta = 627 (95% CI 532–723), *p* = 6.32 × 10^−36^]. We did not find support for a clinical impact of this SNP in terms of association with risk of bleeding or thromboembolic events.

Previous small studies (Dimatteo et al., 2016; Lahteenmaki et al., 2021) have shown that the minor allele of rs4148738, an expression quantitative trait loci (eQTL) for the efflux transporter gene *ABCB1* (Shou et al., 2012), was associated with lower apixaban peak concentrations (Dimatteo et al., 2016) and a lower risk of bleeding events in apixaban users (Lahteenmaki et al., 2021). However, these results could not be confirmed in our larger study. Neither did we replicate previous findings of an association between *CYP3A5* and the PK of apixaban (Ueshima et al., 2017; Ueshima et al., 2018). These discrepancies could probably be explained by our more robust estimates of apixaban exposure, larger sample size, and an adequately powered study.

A limitation of this study is that most participants were of white European ancestry, and thus the results cannot be generalised to all ancestries. Another limitation is the sparseness of the PK data, which may cause shrinkage in the individual parameter estimates (empirical Bayes estimates, EBEs) and thereby an underestimation of the true variability in apixaban exposure. However, we estimated the shrinkage to be less than 15% in this patient cohort, suggesting that this has not impacted the results (Savic and Karlsson, 2009). A further limitation is the small number of clinical events, especially for the stroke categories, which leads to low power to detect a genetic effect.

## 6 Conclusion

This study demonstrates that genetic variation in the drug transporter gene *ABCG2* affects the pharmacokinetics of apixaban. Patients carrying the *ABCG2* variant rs2231142 were exposed to higher concentrations of apixaban [AUC, beta = 151 (95% CI 59–243), *p* = 0.001]. The effect of rs2231142 did not appear to translate into an increased risk of bleeding or thromboembolic events, although the number of bleeding events were low, and the results should be interpreted with caution. Further studies are needed to better understand the occurrence of ischemic and bleeding events during the use of apixaban.

## Data Availability

The data analyzed in this study is subject to the following licenses/restrictions: The datasets generated during and/or analysed during the current study are not publicly available due to the European General Data Protection Regulation (GDPR), which requires us to protect the identity of participants, but datasets are partly available from the corresponding author on reasonable request. Requests to access these datasets should be directed to Mia.Wadelius@medsci.uu.se; Niclas.Eriksson@ucr.uu.se.
