# Supplementary material for: Genetic determinants of apixaban plasma levels and their relationship to bleeding and thromboembolic events
Source: Front Genet. 2022 Sep 14;13:982955. doi: 10.3389/fgene.2022.982955 (PMC9515473; doi:10.3389/fgene.2022.982955)
Supplement: Supplementary file 1 [file DataSheet1.docx]

Supplementary Material

Contents

[**Nominally significant SNPs in GWAS** 3](#_Toc110070504)

[**Supplementary Figure 1.** Population structure compared to HapMap of all 2,800 apixaban treated patients. 4](#_Toc110070505)

[**Supplementary Figure 2.** Principal component analysis (PCA) plot divided by sensitivity groups. 5](#_Toc110070506)

[**Supplementary Figure 3.** Power versus the R^2^ of a genetic marker for the GWAS using 1,325 observations versus a continuous trait at the genome wide significance level of 5*10-8 6](#_Toc110070507)

[**Supplementary Figure 4.** Estimated power versus varying odds ratio and MAF for an analysis of a clinical outcome with total number of observations 2,799 and 105 events 7](#_Toc110070508)

[**Supplementary Figure 5.** Estimated power versus varying odds ratio and MAF for an analysis of a clinical outcome with total number of observations 2,799 and 214 events 8](#_Toc110070509)

[**Supplementary Figure 6**. Manhattan plot for the GWAS of apixaban C_max,ss_ 9](#_Toc110070510)

[**Supplementary Figure 7.** Manhattan plot for the GWAS of apixaban C_min,ss_ 10](#_Toc110070511)

[**Supplementary Figure 8.** Sensitivity analysis in patients of mainly white ethnicity (N=1,174) according to the principal component analysis - Manhattan plot for the GWAS of apixaban AUC_ss_ 11](#_Toc110070512)

[**Supplementary Figure 9**. Sensitivity analysis in patients of mainly white ethnicity (N=1,174) according to the principal component analysis - Manhattan plot for the GWAS of apixaban C_max,ss_ 12](#_Toc110070513)

[**Supplementary Figure 10.** Sensitivity analysis in patients of mainly white ethnicity (N=1,174) according to the principal component analysis - Manhattan plot for the GWAS of apixaban C_min,ss_ 13](#_Toc110070514)

[**Supplementary Table 1.** Descriptives of clinical outcomes for patients treated with apixaban 14](#_Toc110070515)

[**Supplementary Table 2.** List of candidate genes and their genetic positions 15](#_Toc110070516)

[**Supplementary Table 3**. Top 60 variants for the GWAS of apixaban AUC_ss_ 16](#_Toc110070517)

[**Supplementary Table 4**. Top 60 variants for the GWAS of apixaban C_max.ss_ 18](#_Toc110070518)

[**Supplementary Table 5.** Top 60 variants for the GWAS of apixaban C_min.ss_ 20](#_Toc110070519)

[**Supplementary Table 6.** Linear regression result for rs2231142 versus apixaban PK parameters adjusted for clinical factors 22](#_Toc110070520)

[**Supplementary Table 7.** Top ten SNPs per apixaban PK parameter in the candidate gene analyses, adjusted for the covariates listed in Table 3. 23](#_Toc110070521)

[**Supplementary Table 8.** Top 60 variants for the candidate gene analyses of apixaban AUC_ss_ 24](#_Toc110070522)

[**Supplementary Table 9.** Top 60 variants for the candidate gene analyses of apixaban C_max.ss_ 26](#_Toc110070523)

[**Supplementary Table 10.** Top 60 variants for the candidate gene analyses of apixaban C_min.ss_ 28](#_Toc110070524)

[**Supplementary Table 11.** Table of clinical outcomes by levels of rs2231142 30](#_Toc110070525)

[**References** 31](#_Toc110070526)

### **Nominally significant SNPs in GWAS**

*Nominally significant SNPs associated with apixaban AUC_ss_*

rs111844911 is located within an intron or 5’UTR on chromosome 7 in the Fascin Actin-bundling protein 1 gene (FSCN1). FSCN1 encodes a member of the Fascin family of Actin-binding proteins, which are required for the formation of actin-based cellular protrusions, and is involved in cell migration, motility, adhesion and cellular interactions [1]. This SNP is located within a region with evidence for altered chromatin state and histone modification in a wide variety of cell lines [2].

rs183109587 and rs184794076 are located in a genetic desert on chromosome X and both have a very low MAF (0.01). The closest neighbouring gene DDB1 and CUL4 associated factor 12 like 2 (DCAF12L2) encodes a member of the WD repeat protein family [3]. WD proteins are involved in a variety of cellular processes, including cell cycle progression, signal transduction, apoptosis and gene regulation. rs183109587 has no evidence for a regulatory function [2].

rs59884489 is an intergenic SNP close to the Cerebellin 1 precursor gene (CBLN1) on chromosome 16. This gene encodes precerebellin, which is a cerebellum-specific precursor protein that is processed to, e.g., cerebellin [4]. Cerebellin is highly enriched in postsynaptic structures of Purkinje cells. rs59884489 is in LD (r^2^>0.8) with several SNPs within regions with evidence for altered chromatin state and histone modification in e.g., brain, muscle and gastrointestinal cell lines [2].

*Nominally significant SNPs associated with apixaban Cmax_ss_*

rs56293342 is an intergenic SNP located on chromosome 2 near the T-box brain transcription factor 1 gene (TBR1). This gene encodes a transcription factor involved in the regulation of numerous developmental processes and is highly conserved [5]. This SNP is located within a region with evidence for altered chromatin state and histone modification in a wide variety of cell lines [2].

*Nominally significant SNPs associated with apixaban Cmin_ss_*

rs78896694 is an intergenic SNP located on chromosome 7 and has a very low MAF (0.012). Its closest neighbouring gene is Diacylglycerol Kinase Beta (DGKB) located 43k bases away. Diacylglycerol Kinases are regulators of the intracellular concentration of the second messenger diacylglycerol [6]. rs78896694 is located within a region with very little evidence for altered chromatin state and histone modification [2].

rs143178045 is an intronic SNP in the Sarcoglycan Zeta (SGCZ) gene located on chromosome 8 and has a very low MAF (0.014). This gene encodes a protein which is part of the sarcoglycan complex [7]. Sarcoglycans are transmembrane proteins, and the sarcoglycan complex is part of the Dystrophin-associated Glycoprotein Complex (DGC), which bridges the inner cytoskeleton and the extra-cellular matrix. rs143178045 has no evidence for a regulatory function [2].

rs12107681 is an intronic SNP in a gene that is transcribed into non-coding RNA (LOC105374016), located on chromosome 3. It is located within a region with very little evidence for altered chromatin state and histone modification [2].

rs7608316 is an intergenic SNP on chromosome 2. Its closest neighbouring gene is Long Intergenic Non-Protein Coding RNA 1797 (LINC01797) located 35k bases away. rs7608316 is located within a region with evidence for altered chromatin state and histone modification in a variety of tissues, e.g., neurons, chondrocytes, fibroblasts, skin, smooth muscle, kidney and lung [2].

No SNP putatively associated (p<1x10^-6^) with AUCss (rs111844911, rs183109587, rs184794076, and rs59884489), Cmin,ss (rs147256925, rs78896694, rs143178045, and rs12107681) or Cmax,ss (rs56293342) is a known expression quantitative trait loci (eQTL) for a protein coding gene according to the EBI eQTL catalogue [8]. The SNP putatively associated with Cmax,ss (rs56293342) is an eQTL for the Long Intergenic Non-Protein Coding RNA 1806 (LINC01806) in skin with an effect size of 0.89 (p= 1x10^-9.62^). The clinical importance of this is unknown.

### **Supplementary Figure 1.** Population structure compared to HapMap of all 2,800 apixaban treated patients.

Plot of the first two genetic principal components. HapMap population samples are CEU = Utah residents with Northern and Western European ancestry from the CEPH collection. CHB = Han Chinese in Beijing (China). JPT = Japanese in Tokyo (Japan). YRI = Yoruba in Ibadan (Nigeria). PC = principal component.

### **Supplementary Figure 2.** Principal component analysis (PCA) plot divided by sensitivity groups.

Plot of genetic principal components 1 and 2 divided by patients in sensitivity analysis group (right panel, 1,174 patients of mainly white self-reported ethnicity) and excluded group (left panel).


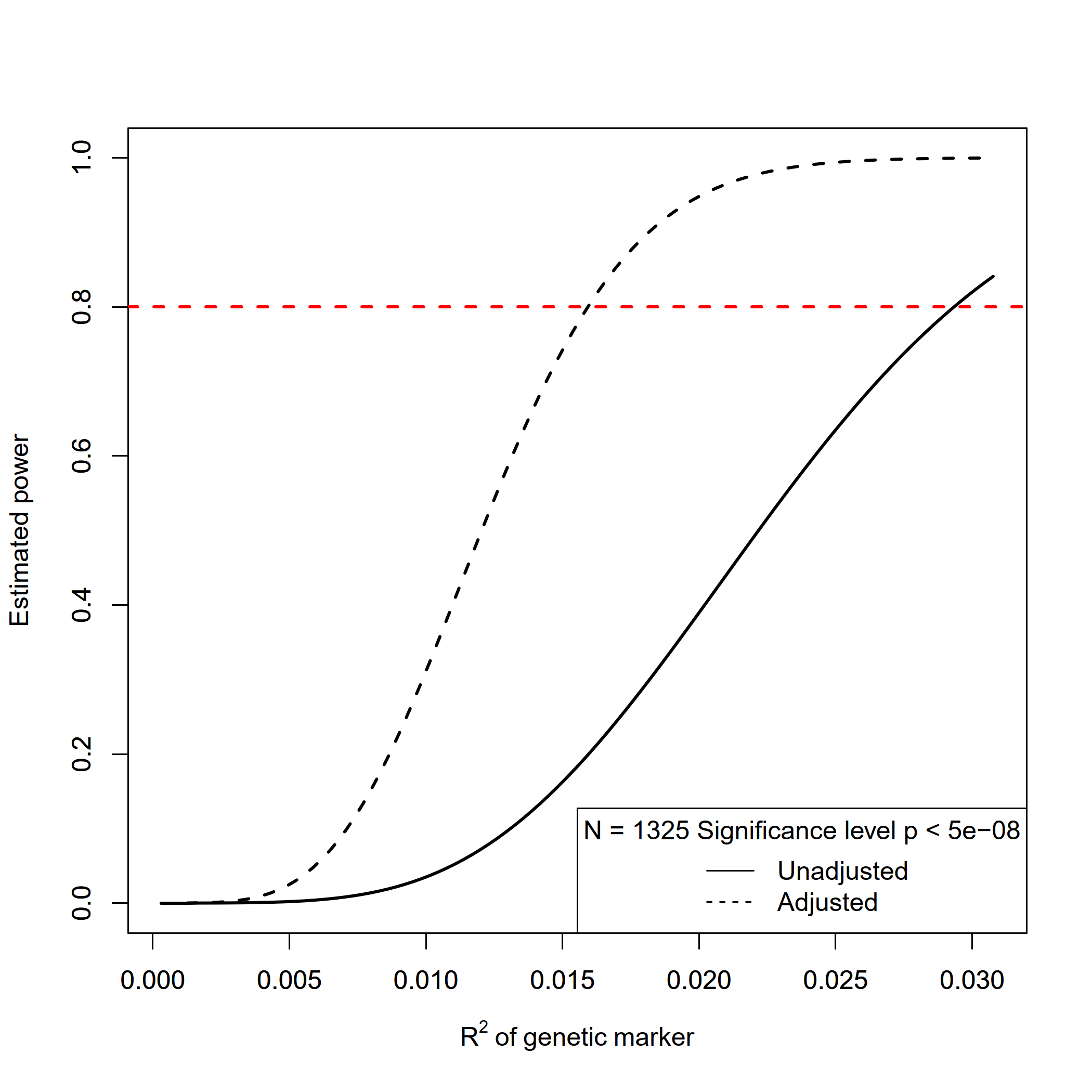


**Supplementary Figure 3.** Power versus the R^2^ of a genetic marker for the GWAS using 1,325 observations versus a continuous trait at the genome wide significance level of 5*10-8. The solid line indicates the power for an unadjusted model whereas the dashed line indicates the power when adjusting for covariates explaining 0.458 of the variance.


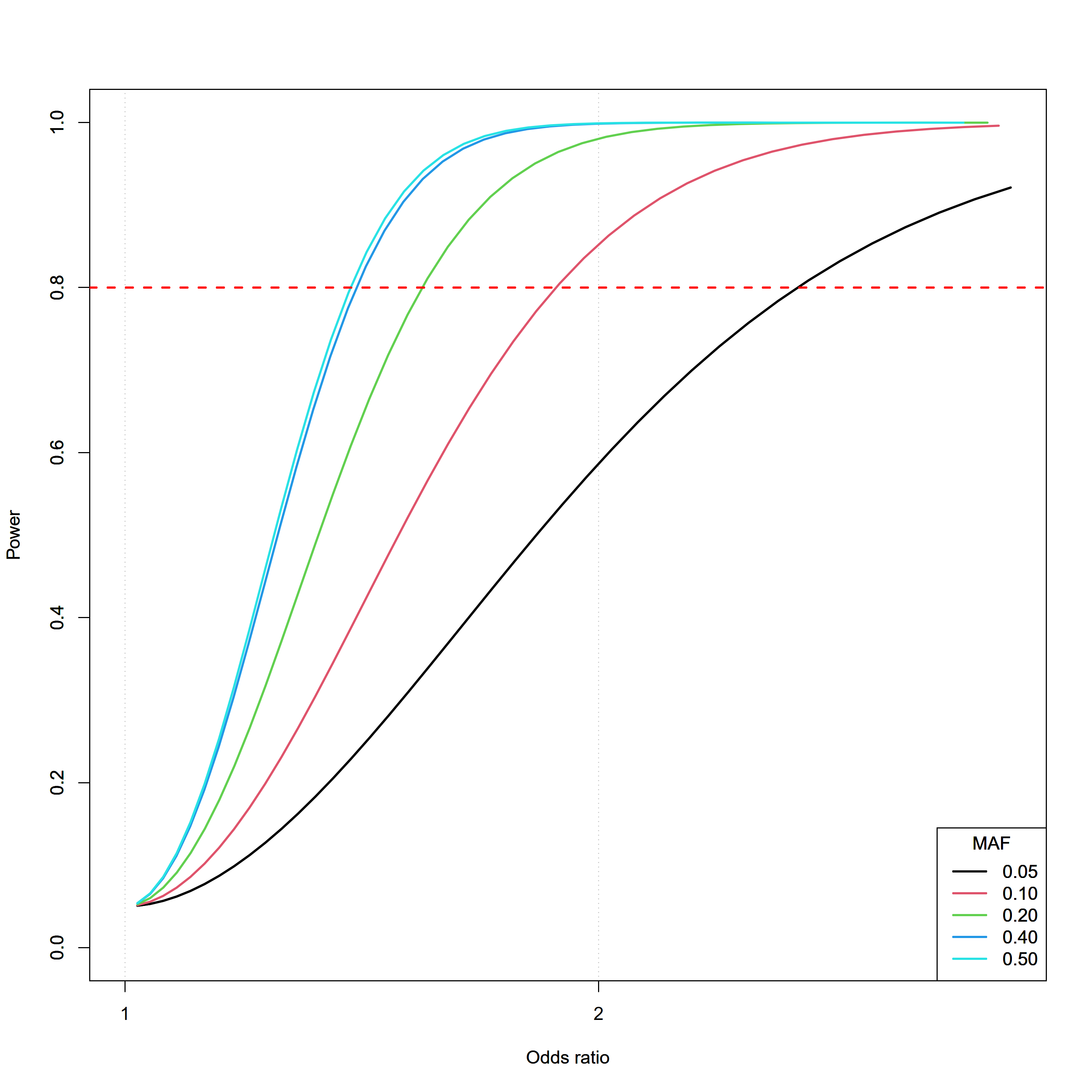


**Supplementary Figure 4.** Estimated power versus varying odds ratio and MAF for an analysis of a clinical outcome with total number of observations 2,799 and 105 events (corresponding to the number of major bleeding events in this paper). This power analysis has an alpha level of 0.05 i.e. it has no adjustment for multiple tests.


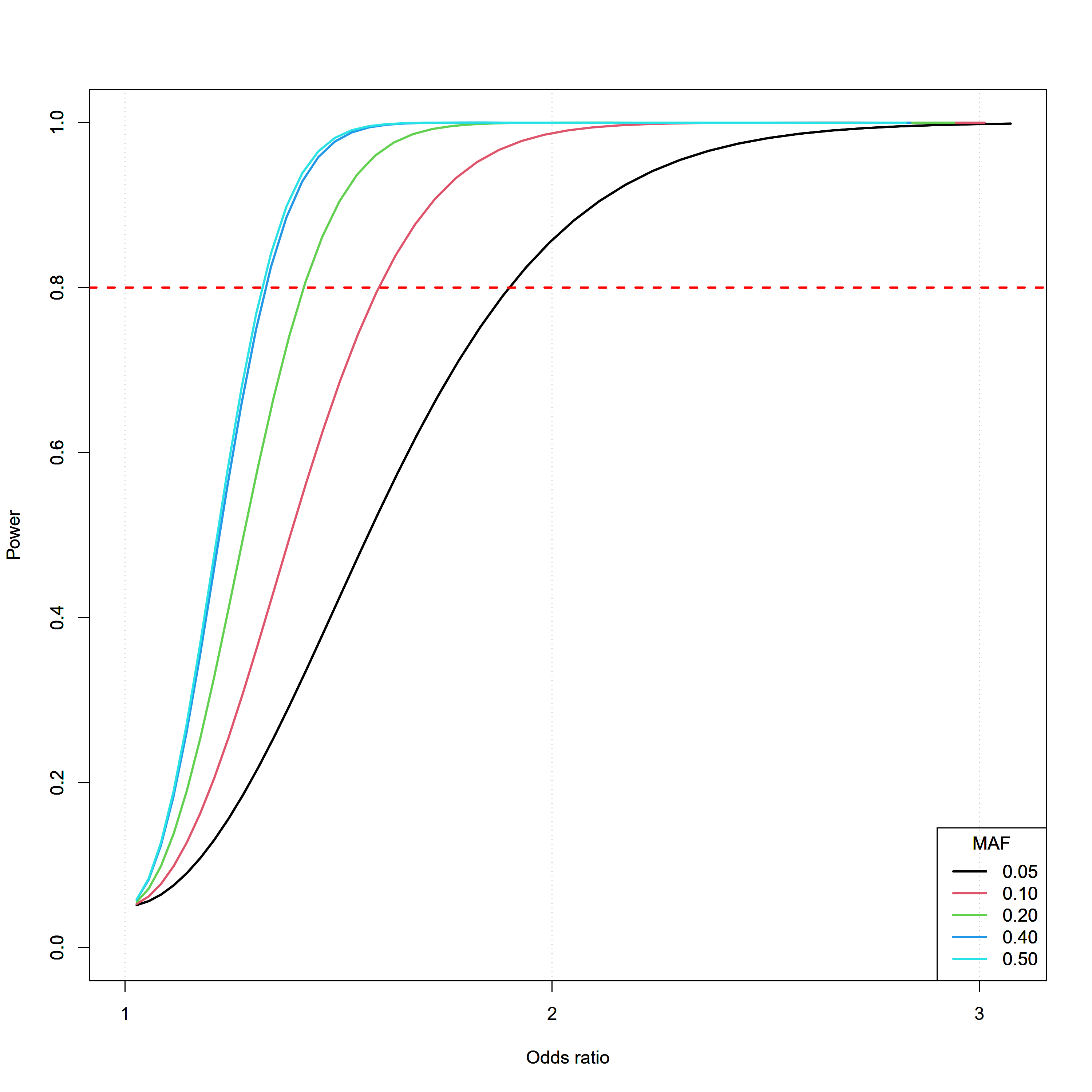


**Supplementary Figure 5.** Estimated power versus varying odds ratio and MAF for an analysis of a clinical outcome with total number of observations 2,799 and 214 events (corresponding to the number of Major/Clinically relevant non-major bleeding events in this paper). This power analysis has an alpha level of 0.05 i.e. it has no adjustment for multiple tests.


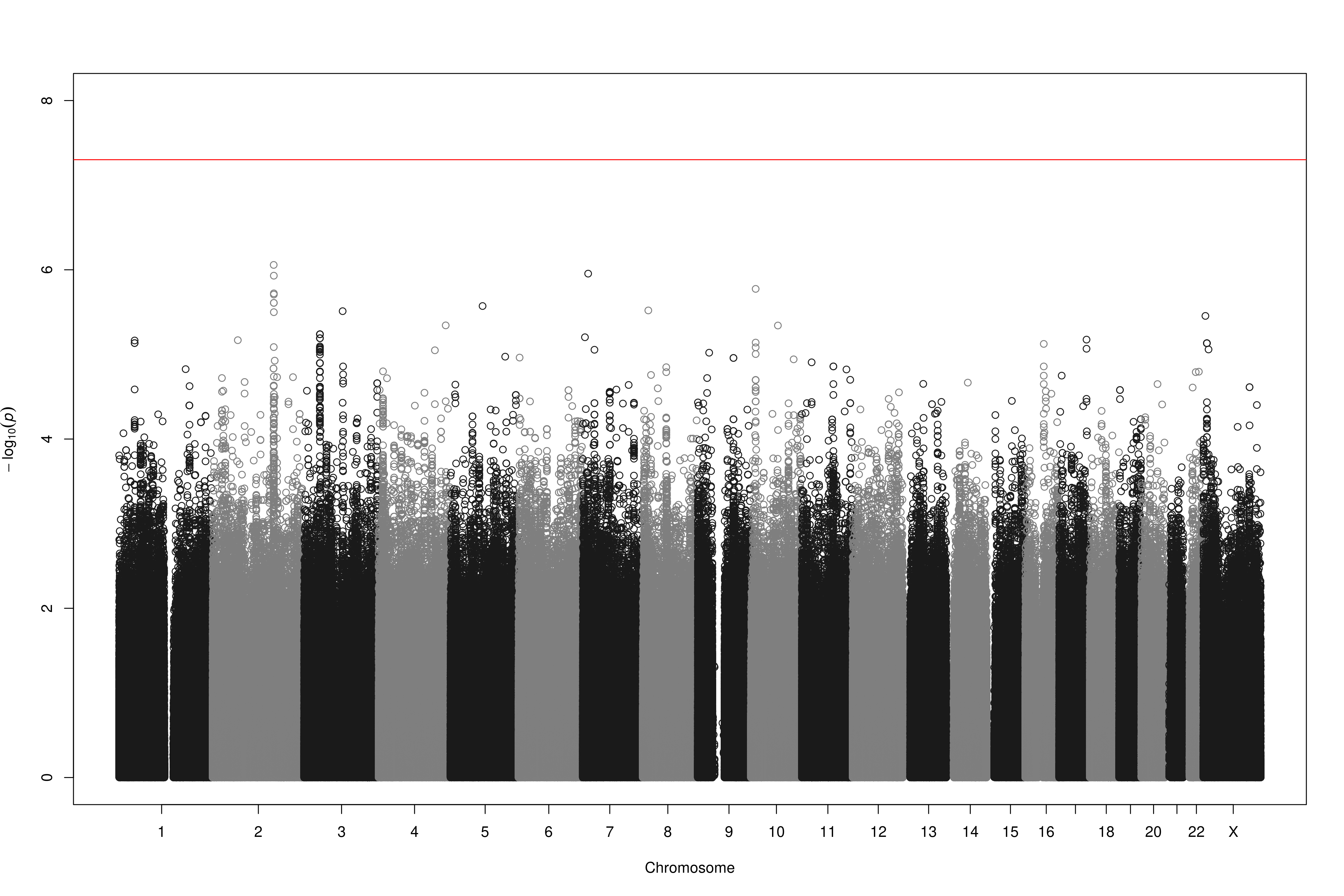


**Supplementary Figure 6**. Manhattan plot for the GWAS of apixaban C_max,ss_ in 1,325 patients* The grey line denotes the genome-wide significance level p <5x10^-8^.

*Adjustment was done for the covariates listed in Table 3 in the main manuscript.


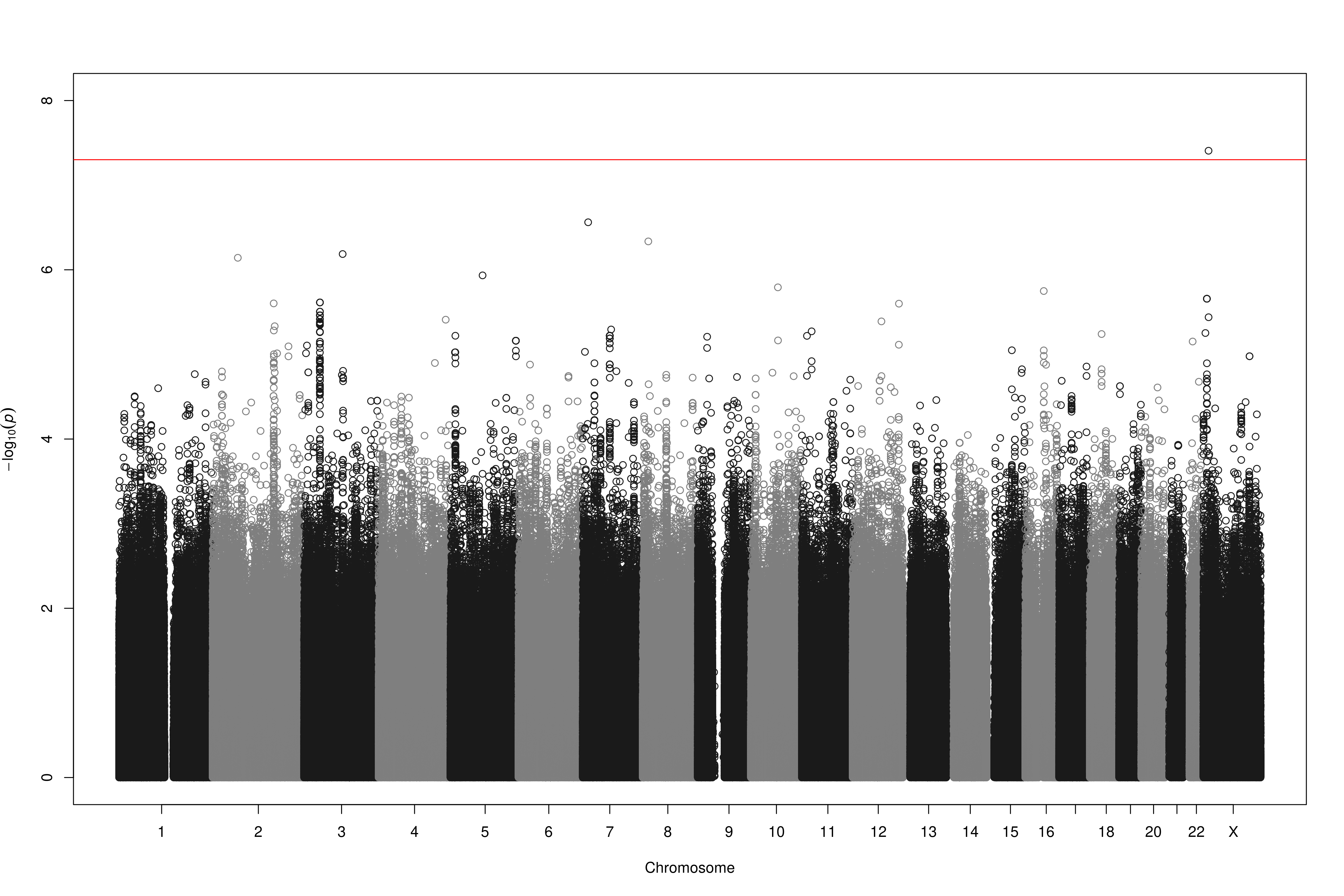


**Supplementary Figure 7.** Manhattan plot for the GWAS of apixaban C_min,ss_ in 1,325 patients* The grey line denotes the genome-wide significance level p <5x10^-8^.

*Adjustment was done for the covariates listed in Table 3 in the main manuscript.


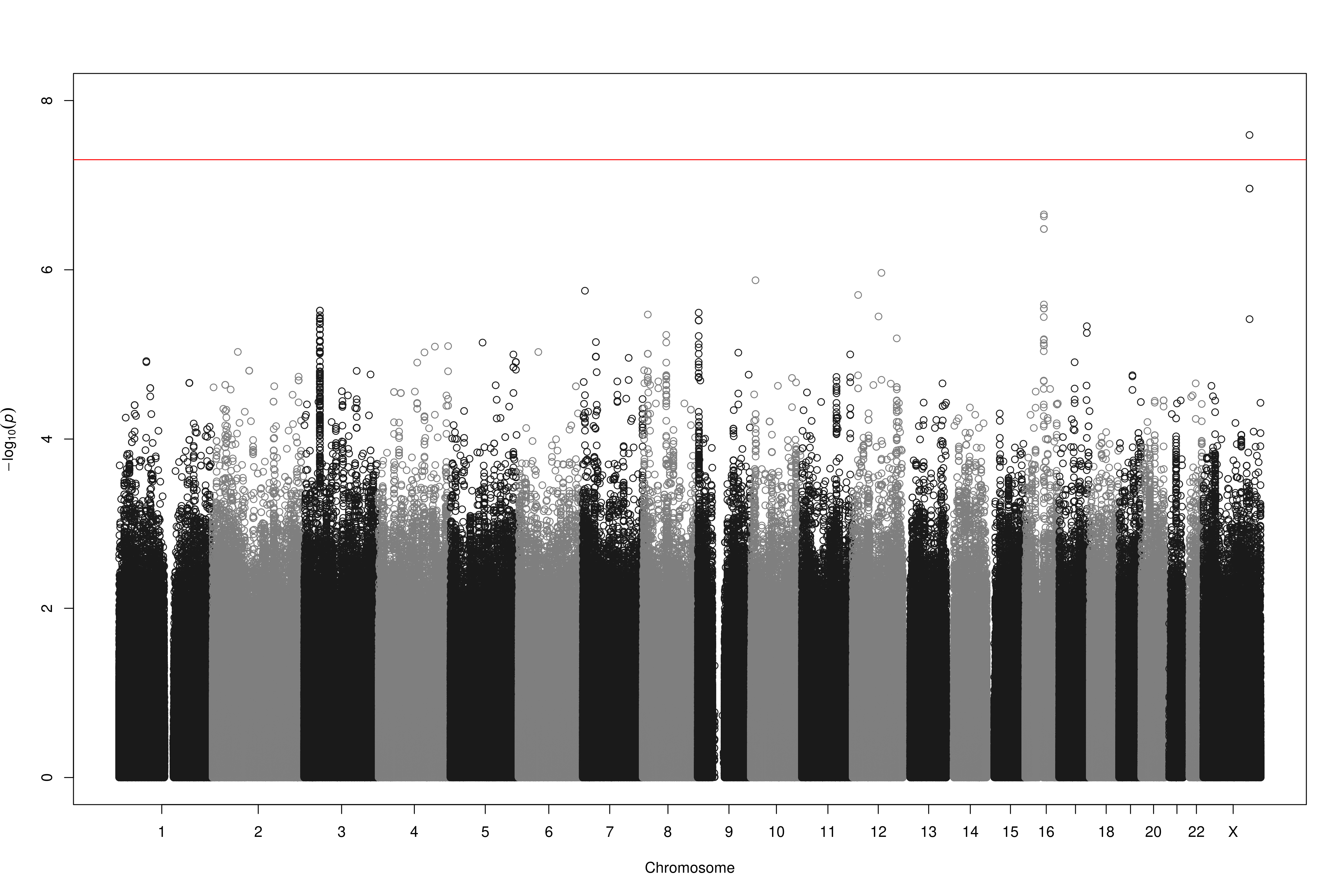


**Supplementary Figure 8.** Sensitivity analysis in patients of mainly white ethnicity (N=1,174) according to the principal component analysis - Manhattan plot for the GWAS of apixaban AUC_ss_* The grey line denotes the genome-wide significance level p <5x10^-8^.

*Adjustment was done for the covariates listed in Table 3 in the main manuscript.


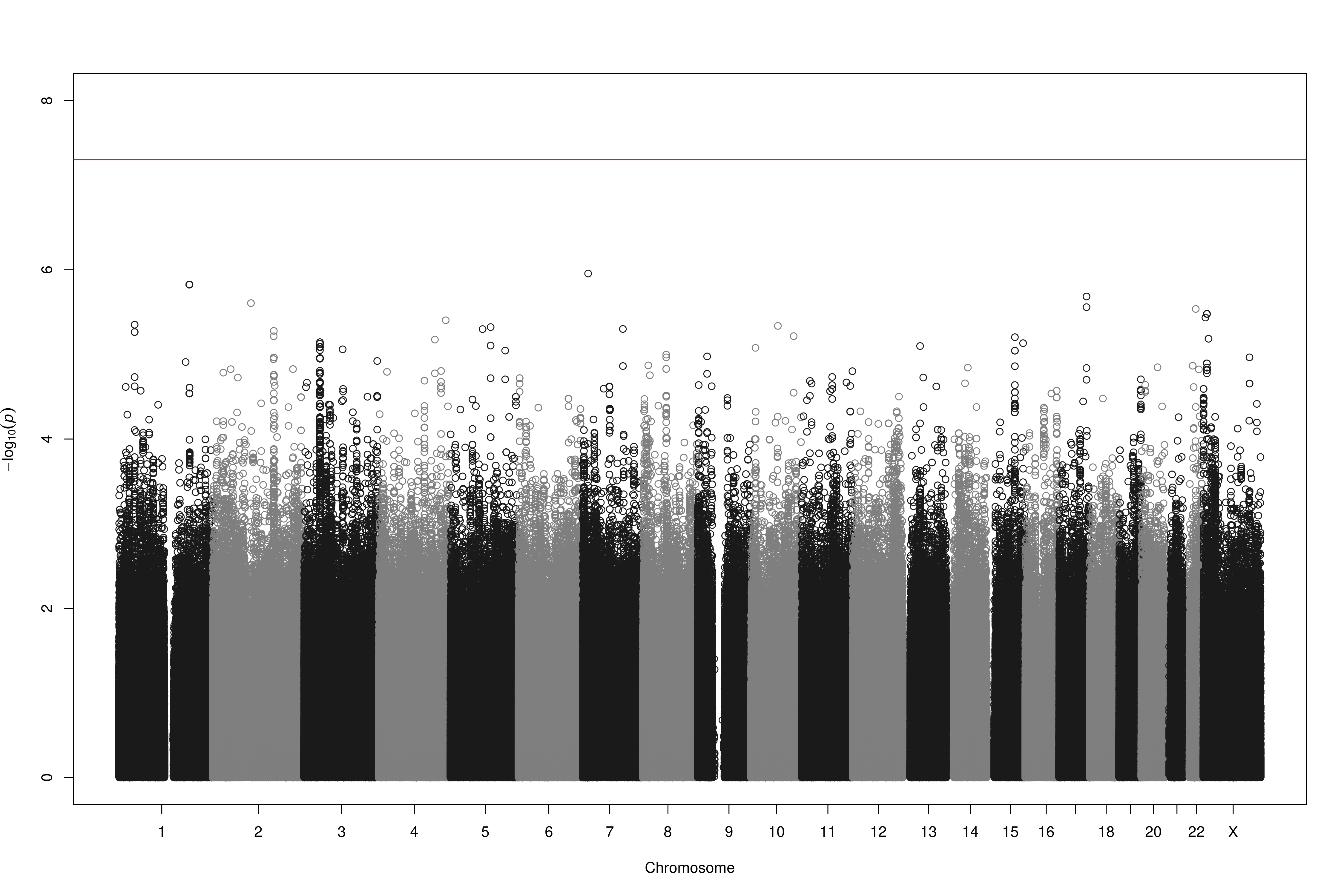


**Supplementary Figure 9**. Sensitivity analysis in patients of mainly white ethnicity (N=1,174) according to the principal component analysis - Manhattan plot for the GWAS of apixaban C_max,ss_* The grey line denotes the genome-wide significance level p <5x10^-8^.

*Adjustment was done for the covariates listed in Table 3 in the main manuscript.


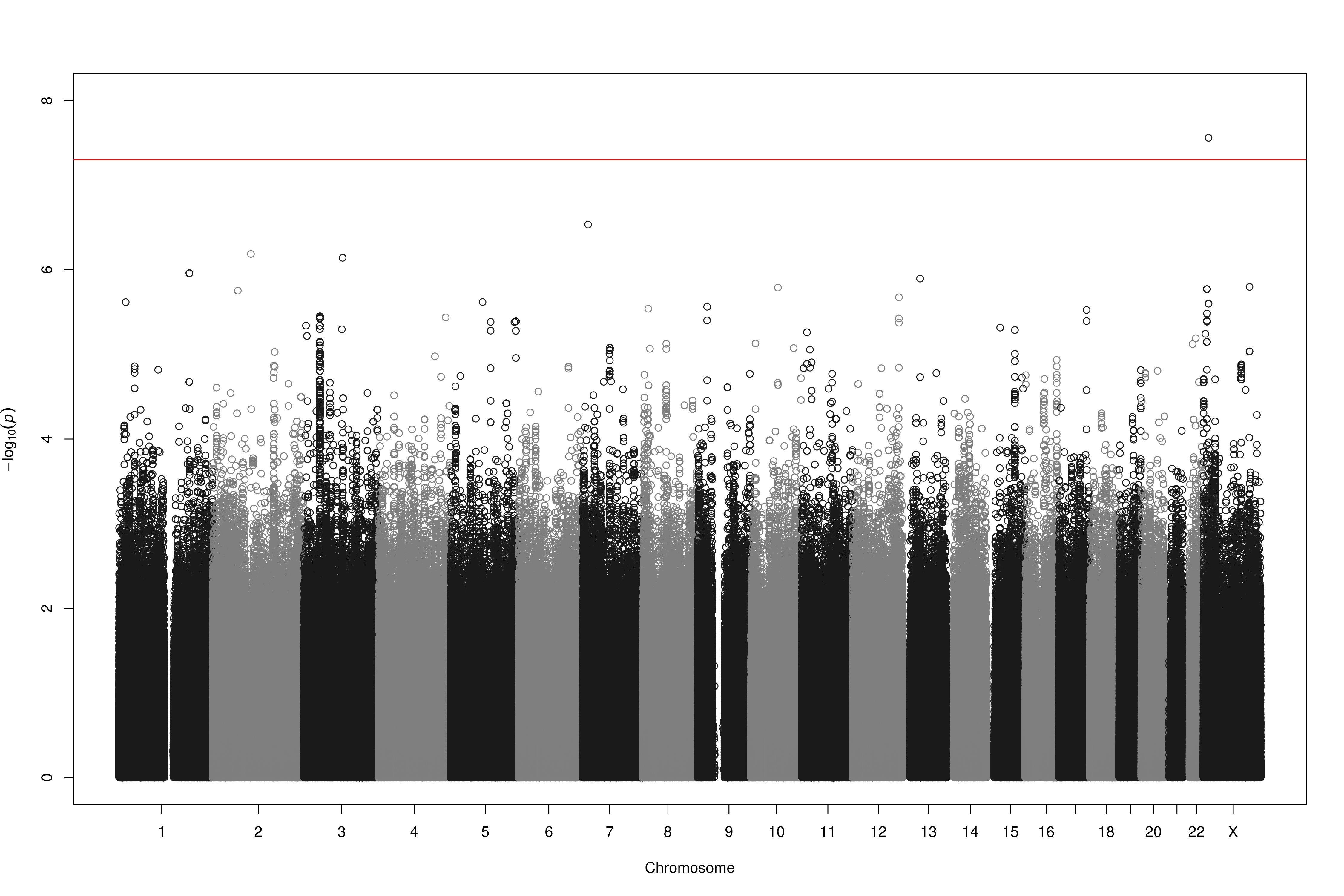


**Supplementary Figure 10.** Sensitivity analysis in patients of mainly white ethnicity (N=1,174) according to the principal component analysis - Manhattan plot for the GWAS of apixaban C_min,ss_ * The grey line denotes the genome-wide significance level p <5x10^-8^.

*Adjustment was done for the covariates listed in Table 3 in the main manuscript.

### **Supplementary Table 1.** Descriptives of clinical outcomes for patients treated with apixaban

|  | | | | | | |
| --- | --- | --- | --- | --- | --- | --- |
| **Outcome** | **N** | **N events** | **Median follow-up (years)** | **Max follow-up (years)** | **Patient years** | **Incidence rate (CI) ¤** |
| **Major bleeding (ISTH)** | 2799 | 105 | 1.82 | 3.93 | 5123.61 | 2.05 (1.68 - 2.48) |
| **Major / CRNM bleeding (ISTH)** | 2799 | 214 | 1.80 | 3.93 | 5025.54 | 4.26 (3.71 - 4.87) |
| **Stroke / Systemic embolism** | 2800 | 54 | 1.89 | 4.04 | 5478.27 | 0.99 (0.74 - 1.29) |
| **Haemorrhagic stroke** | 2800 | 11 | 1.89 | 4.04 | 5511.88 | 0.20 (0.10 - 0.36) |
| **Ischemic stroke** | 2800 | 32 | 1.89 | 4.04 | 5489.75 | 0.58 (0.40 - 0.82) |

¤ Incidence rate calculated per 100 patient years. The 95 percent confidence interval (CI) for the incidence rate was estimated using a gamma distribution.

N = number of patients with available event data. N events = number of observed events. ISTH = International Society on Thrombosis and Haemostasis; CRNM = clinically relevant non-major.

### **Supplementary Table 2.** List of candidate genes and their genetic positions

| **Gene** | **Chromosome** | **Start Position** | **End Position** |
| --- | --- | --- | --- |
| SULT1A1 [9] | 16 | 28616907 | 28620649 |
| ABCB1 [10, 11] | 7 | 87133178 | 87342639 |
| ABCG2 [12] | 4 | 89011415 | 89080011 |
| CYP3A5 [12, 10] | 7 | 99245813 | 99277636 |
| CYP3A4 [13] | 7 | 99354582 | 99381811 |

SNP = single nucleotide polymorphism.

### **Supplementary Table 3**. Top 60 variants for the GWAS of apixaban AUC_ss_

| **CHR** | **SNP** | **BP** | **N** | **BETA** | **L95** | **U95** | **P** | **MAF** | **GTPS** | **Gene** |  |
| --- | --- | --- | --- | --- | --- | --- | --- | --- | --- | --- | --- |
| 7 | rs111844911 | 5633530 | 1325 | 462.2 | 296.2 | 628.2 | 5.796E-08 | 0.032 | A/G | FSCN1 |  |
| X | rs183109587 | 125253933 | 1325 | 972.2 | 613.4 | 1331 | 1.285E-07 | 0.01 | T/G |  |  |
| X | rs184794076 | 125268644 | 1325 | 972.2 | 613.4 | 1331 | 1.285E-07 | 0.01 | C/A |  |  |
| 16 | rs59884489 | 49252034 | 1325 | 252.8 | 158.3 | 347.3 | 1.833E-07 | 0.103 | T/C |  |  |
| X | rs144905736 | 125310708 | 1325 | 749.7 | 449.9 | 1049 | 1.072E-06 | 0.015 | C/A |  |  |
| 16 | rs8056754 | 49232352 | 1325 | 224.5 | 134.3 | 314.7 | 1.213E-06 | 0.119 | C/T |  |  |
| 16 | rs72776712 | 49215955 | 1325 | 222.5 | 132.2 | 312.7 | 1.509E-06 | 0.119 | T/C |  |  |
| 9 | rs872628 | 107815188 | 1325 | -150.6 | -211.9 | -89.3 | 1.639E-06 | 0.361 | A/G |  |  |
| 5 | rs76518546 | 120106043 | 1325 | -476.8 | -671.8 | -281.8 | 1.841E-06 | 0.023 | A/G |  |  |
| 16 | rs12446500 | 49212965 | 1325 | 220.3 | 130.2 | 310.5 | 1.854E-06 | 0.12 | T/G |  |  |
| 10 | rs12569927 | 12789892 | 1325 | -174.4 | -246.1 | -102.7 | 0.00000208 | 0.21 | G/A | CAMK1D |  |
| 12 | rs7963744 | 14650072 | 1325 | 188.6 | 110.9 | 266.3 | 2.184E-06 | 0.177 | C/T | ATF7IP |  |
| 10 | rs17152197 | 12792541 | 1325 | -181.2 | -255.9 | -106.5 | 2.197E-06 | 0.194 | C/G | CAMK1D |  |
| 11 | rs1148074 | 128596898 | 1325 | -253.6 | -358.3 | -149 | 2.243E-06 | 0.083 | G/A | FLI1 |  |
| 12 | rs114554346 | 118666918 | 1325 | -662.5 | -935.9 | -389.1 | 2.265E-06 | 0.013 | G/T | TAOK3 |  |
| 12 | rs117943690 | 76590246 | 1325 | 525.3 | 306.8 | 743.8 | 2.709E-06 | 0.018 | T/C |  |  |
| 10 | rs2095566 | 120248621 | 1325 | -162.8 | -230.7 | -94.95 | 2.861E-06 | 0.275 | A/G |  |  |
| 7 | rs117053447 | 5634203 | 1325 | 374.5 | 218.2 | 530.8 | 2.924E-06 | 0.037 | A/G | FSCN1 |  |
| 2 | rs79002239 | 163736890 | 1325 | -625.8 | -888.8 | -362.8 | 3.421E-06 | 0.013 | A/G |  |  |
| 10 | rs12570883 | 12789676 | 1325 | -176.5 | -250.8 | -102.1 | 3.617E-06 | 0.196 | A/G | CAMK1D |  |
| 2 | rs7608316 | 66957093 | 1325 | 570.9 | 330.1 | 811.7 | 3.711E-06 | 0.015 | G/A |  |  |
| 7 | rs3801004 | 5638222 | 1325 | 239.1 | 138.1 | 340.1 | 0.00000382 | 0.096 | G/C | FSCN1 |  |
| 1 | rs2292822 | 201777568 | 1325 | 617.5 | 356.6 | 878.3 | 3.841E-06 | 0.015 | G/C | NAV1 |  |
| 11 | rs646271 | 128611160 | 1325 | -245.3 | -349.2 | -141.4 | 4.095E-06 | 0.084 | T/C | FLI1 |  |
| 5 | rs62364239 | 85293185 | 1325 | -305.2 | -434.9 | -175.6 | 4.344E-06 | 0.057 | T/G |  |  |
| 9 | rs7875832 | 107791221 | 1325 | -142.5 | -203.1 | -81.78 | 0.0000046 | 0.373 | T/C |  |  |
| 13 | rs12586000 | 102172425 | 1325 | -160.1 | -228.3 | -91.82 | 4.687E-06 | 0.263 | T/C | ITGBL1 |  |
| 11 | rs77788210 | 128613641 | 1325 | -243.3 | -347 | -139.5 | 4.749E-06 | 0.084 | A/G | FLI1 |  |
| 2 | rs13026747 | 162364504 | 1325 | 186.9 | 107.1 | 266.7 | 4.801E-06 | 0.162 | G/T |  |  |
| 2 | rs56240304 | 33558778 | 1325 | 437.9 | 250.7 | 625.1 | 4.978E-06 | 0.025 | G/A | LTBP1 |  |
| 2 | rs77969817 | 33562637 | 1325 | 437.9 | 250.7 | 625.1 | 4.978E-06 | 0.025 | A/G | LTBP1 |  |
| 3 | 3:42004427 | 42004427 | 1325 | 197.1 | 112.9 | 281.4 | 4.981E-06 | 0.145 | C/A |  |  |
| 3 | rs113379006 | 42008186 | 1325 | 197.1 | 112.9 | 281.4 | 4.981E-06 | 0.145 | T/C |  |  |
| 3 | rs114478966 | 42007050 | 1325 | 197.1 | 112.9 | 281.4 | 4.981E-06 | 0.145 | C/T |  |  |
| 3 | rs55953604 | 42004428 | 1325 | 197.1 | 112.9 | 281.4 | 4.981E-06 | 0.145 | G/A |  |  |
| 2 | rs79318988 | 33528001 | 1325 | 409.4 | 234.1 | 584.6 | 5.145E-06 | 0.028 | A/G | LTBP1 |  |
| 9 | rs80218026 | 135273932 | 1325 | 588.6 | 336.5 | 840.6 | 5.179E-06 | 0.014 | G/A | TTF1 |  |
| 4 | rs7675722 | 121884592 | 1325 | 201 | 114.8 | 287.1 | 5.269E-06 | 0.132 | G/A |  |  |
| 17 | rs34472315 | 73396830 | 1325 | -508.8 | -727.1 | -290.5 | 5.376E-06 | 0.019 | T/C | GRB2 |  |
| 12 | rs142623524 | 68820434 | 1325 | 528.3 | 301.7 | 755 | 5.379E-06 | 0.018 | T/C |  |  |
| 16 | rs72776706 | 49176247 | 1325 | 240.1 | 137.1 | 343.2 | 5.386E-06 | 0.089 | G/T |  |  |
| 11 | rs145640062 | 128630686 | 1325 | -236.3 | -337.7 | -134.9 | 5.404E-06 | 0.088 | T/G | FLI1 |  |
| 3 | rs113144620 | 42008312 | 1325 | 195.7 | 111.5 | 279.9 | 5.703E-06 | 0.146 | G/A |  |  |
| 3 | rs73073226 | 42005838 | 1325 | 195.7 | 111.5 | 279.9 | 5.703E-06 | 0.146 | C/T |  |  |
| 3 | rs74947509 | 42006323 | 1325 | 195.7 | 111.5 | 279.9 | 5.703E-06 | 0.146 | C/T |  |  |
| 11 | rs7479732 | 92088177 | 1325 | 167.8 | 95.58 | 240 | 0.00000573 | 0.212 | T/C | FAT3 |  |
| 17 | rs36114612 | 73407812 | 1325 | -516 | -738.7 | -293.4 | 6.072E-06 | 0.018 | T/C |  |  |
| 2 | rs10202267 | 162366126 | 1325 | 184.8 | 104.9 | 264.7 | 0.00000632 | 0.16 | T/A |  |  |
| 2 | rs3849353 | 162374138 | 1325 | 184.8 | 104.9 | 264.7 | 0.00000632 | 0.16 | G/C |  |  |
| 2 | rs7588198 | 162365202 | 1325 | 184.8 | 104.9 | 264.7 | 0.00000632 | 0.16 | G/T |  |  |
| 2 | rs7592112 | 162366517 | 1325 | 184.8 | 104.9 | 264.7 | 0.00000632 | 0.16 | C/G |  |  |
| 2 | rs7595197 | 162366835 | 1325 | 184.8 | 104.9 | 264.7 | 0.00000632 | 0.16 | G/T |  |  |
| 10 | rs11813941 | 12790529 | 1325 | -170 | -243.6 | -96.36 | 6.568E-06 | 0.2 | C/T | CAMK1D |  |
| 10 | rs11813942 | 12790535 | 1325 | -170 | -243.6 | -96.36 | 6.568E-06 | 0.2 | A/T | CAMK1D |  |
| 11 | rs1941441 | 92060517 | 1325 | 166.1 | 94.12 | 238.1 | 6.638E-06 | 0.213 | T/C |  |  |
| 10 | rs57865422 | 12795205 | 1325 | -174 | -249.4 | -98.6 | 6.663E-06 | 0.189 | T/C | CAMK1D |  |
| 10 | rs4558057 | 12791605 | 1325 | -173.3 | -248.6 | -97.99 | 7.065E-06 | 0.191 | T/C | CAMK1D |  |
| 10 | rs74118561 | 12791141 | 1325 | -173.3 | -248.6 | -97.99 | 7.065E-06 | 0.191 | A/G | CAMK1D |  |
| 3 | 3:42002761 | 42002761 | 1325 | 194.2 | 109.8 | 278.7 | 7.081E-06 | 0.145 | G/A | ULK4 |  |
| 3 | 3:42008263 | 42008263 | 1325 | 194.2 | 109.8 | 278.7 | 7.081E-06 | 0.145 | A/G | | |

CHR = chromosome; SNP = single nucleotide polymorphism; BP = base pair; L95 = lower 95% confidence interval; U95 = upper 95% confidence interval; MAF = minor allele frequency; GTPS = Guanosine-5′-triphosphates.

### **Supplementary Table 4**. Top 60 variants for the GWAS of apixaban C_max.ss_

| \| **CHR** \| **SNP** \| **BP** \| **N** \| **BETA** \| **L95** \| **U95** \| **P** \| **MAF** \| **GTPS** \| **Gene** \| \| --- \| --- \| --- \| --- \| --- \| --- \| --- \| --- \| --- \| --- \| --- \| \| 2 \| rs56293342 \| 162283618 \| 1325 \| 16.97 \| 10.24 \| 23.7 \| 8.757E-07 \| 0.083 \| G/C \|  \| \| \| 7 \| rs78896694 \| 14142574 \| 1325 \| 40.63 \| 24.36 \| 56.91 \| 0.00000111 \| 0.012 \| T/C \|  \| \| \| 2 \| rs62189057 \| 162630944 \| 1325 \| 16.99 \| 10.17 \| 23.82 \| 1.174E-06 \| 0.08 \| G/A \| SLC4A10 \| \| \| 10 \| rs12569927 \| 12789892 \| 1325 \| -10.92 \| -15.37 \| -6.472 \| 1.681E-06 \| 0.21 \| G/A \| CAMK1D \| \| \| 2 \| rs62187697 \| 162676532 \| 1325 \| 16.51 \| 9.748 \| 23.27 \| 1.898E-06 \| 0.083 \| G/A \| SLC4A10 \| \| \| 2 \| rs3849343 \| 162668466 \| 1325 \| 16.46 \| 9.709 \| 23.21 \| 1.961E-06 \| 0.083 \| A/C \| SLC4A10 \| \| \| 2 \| rs62190666 \| 162645206 \| 1325 \| 16.33 \| 9.565 \| 23.09 \| 2.459E-06 \| 0.083 \| T/C \| SLC4A10 \| \| \| 2 \| rs62190667 \| 162645269 \| 1325 \| 16.33 \| 9.565 \| 23.09 \| 2.459E-06 \| 0.083 \| A/G \| SLC4A10 \| \| \| 5 \| rs62364239 \| 85293185 \| 1325 \| -19.36 \| -27.4 \| -11.31 \| 2.681E-06 \| 0.057 \| T/G \|  \| \| \| 8 \| rs143178045 \| 14924563 \| 1325 \| 38 \| 22.12 \| 53.87 \| 3.021E-06 \| 0.014 \| A/C \| SGCZ \| \| \| 3 \| rs12107681 \| 102370270 \| 1325 \| 12.58 \| 7.318 \| 17.84 \| 3.083E-06 \| 0.156 \| T/C \|  \| \| \| 2 \| rs72879263 \| 162694101 \| 1325 \| 16.21 \| 9.421 \| 23 \| 3.168E-06 \| 0.082 \| T/G \| SLC4A10 \| \| \| X \| rs188411450 \| 7980122 \| 1325 \| 51.77 \| 29.99 \| 73.54 \| 3.508E-06 \| 0.011 \| C/T \|  \| \| \| 4 \| rs151244651 \| 178345957 \| 1325 \| 32.8 \| 18.84 \| 46.76 \| 4.537E-06 \| 0.018 \| A/G \|  \| \| \| 10 \| rs137906597 \| 71866622 \| 1325 \| 30.12 \| 17.3 \| 42.95 \| 4.555E-06 \| 0.021 \| C/T \| H2AFY2 \| AIFM2 \| \| \| 3 \| 3:42004427 \| 42004427 \| 1325 \| 12.16 \| 6.923 \| 17.39 \| 5.779E-06 \| 0.145 \| C/A \|  \| \| \| 3 \| rs113379006 \| 42008186 \| 1325 \| 12.16 \| 6.923 \| 17.39 \| 5.779E-06 \| 0.145 \| T/C \|  \| \| \| 3 \| rs114478966 \| 42007050 \| 1325 \| 12.16 \| 6.923 \| 17.39 \| 5.779E-06 \| 0.145 \| C/T \|  \| \| \| 3 \| rs55953604 \| 42004428 \| 1325 \| 12.16 \| 6.923 \| 17.39 \| 5.779E-06 \| 0.145 \| G/A \|  \| \| \| 7 \| rs111844911 \| 5633530 \| 1325 \| 23.93 \| 13.59 \| 34.27 \| 6.268E-06 \| 0.032 \| A/G \| FSCN1 \| \| \| 3 \| rs17284472 \| 41996304 \| 1325 \| 12.08 \| 6.851 \| 17.3 \| 6.438E-06 \| 0.146 \| A/G \| ULK4 \| \| \| 3 \| rs6805140 \| 41995791 \| 1325 \| 12.08 \| 6.851 \| 17.3 \| 6.438E-06 \| 0.146 \| G/A \| ULK4 \| \| \| 3 \| rs6807562 \| 41995655 \| 1325 \| 12.08 \| 6.851 \| 17.3 \| 6.438E-06 \| 0.146 \| C/T \| ULK4 \| \| \| 17 \| rs112064953 \| 72625597 \| 1325 \| 26.8 \| 15.18 \| 38.42 \| 6.686E-06 \| 0.026 \| T/C \|  \| \| \| 2 \| rs7608316 \| 66957093 \| 1325 \| 34.48 \| 19.52 \| 49.43 \| 6.789E-06 \| 0.015 \| G/A \|  \| \| \| 1 \| rs12751675 \| 41955714 \| 1325 \| 10.18 \| 5.761 \| 14.59 \| 6.845E-06 \| 0.228 \| A/G \|  \| \| \| 10 \| rs17152197 \| 12792541 \| 1325 \| -10.67 \| -15.31 \| -6.025 \| 7.248E-06 \| 0.194 \| C/G \| CAMK1D \| \| \| 1 \| rs4660541 \| 41954444 \| 1325 \| 9.753 \| 5.507 \| 14 \| 7.346E-06 \| 0.25 \| T/C \|  \| \| \| 1 \| rs71648550 \| 41955090 \| 1325 \| 9.753 \| 5.507 \| 14 \| 7.346E-06 \| 0.25 \| G/A \|  \| \| \| X \| rs28775972 \| 11874988 \| 1325 \| 37.02 \| 20.9 \| 53.13 \| 0.00000737 \| 0.02 \| G/C \|  \| \| \| X \| rs28778135 \| 11872539 \| 1325 \| 37.02 \| 20.9 \| 53.13 \| 0.00000737 \| 0.02 \| G/A \|  \| \| \| X \| rs5979480 \| 11870942 \| 1325 \| 37.02 \| 20.9 \| 53.13 \| 0.00000737 \| 0.02 \| G/C \|  \| \| \| X \| rs6640800 \| 11871811 \| 1325 \| 37.02 \| 20.9 \| 53.13 \| 0.00000737 \| 0.02 \| T/C \|  \| \| \| X \| rs7888276 \| 11868506 \| 1325 \| 37.02 \| 20.9 \| 53.13 \| 0.00000737 \| 0.02 \| G/A \|  \| \| \| 16 \| rs59884489 \| 49252034 \| 1325 \| 13.49 \| 7.612 \| 19.38 \| 7.512E-06 \| 0.103 \| T/C \|  \| \| \| 3 \| rs73071238 \| 41992243 \| 1325 \| 11.96 \| 6.729 \| 17.19 \| 0.00000804 \| 0.146 \| G/A \| ULK4 \| \| \| 2 \| rs62187654 \| 162470969 \| 1325 \| 15.59 \| 8.768 \| 22.42 \| 8.191E-06 \| 0.08 \| T/C \|  \| \| \| 10 \| rs7897966 \| 12793038 \| 1325 \| -10.06 \| -14.46 \| -5.654 \| 8.249E-06 \| 0.214 \| T/C \| CAMK1D \| \| \| 3 \| 3:42002761 \| 42002761 \| 1325 \| 11.97 \| 6.725 \| 17.21 \| 8.327E-06 \| 0.145 \| G/A \| ULK4 \| \| \| 3 \| 3:42008263 \| 42008263 \| 1325 \| 11.97 \| 6.725 \| 17.21 \| 8.327E-06 \| 0.145 \| A/G \|  \| \| \| 3 \| rs10510732 \| 42007339 \| 1325 \| 11.97 \| 6.725 \| 17.21 \| 8.327E-06 \| 0.145 \| A/G \|  \| \| \| 3 \| rs60966614 \| 42005166 \| 1325 \| 11.97 \| 6.725 \| 17.21 \| 8.327E-06 \| 0.145 \| G/A \|  \| \| \| 3 \| rs73071259 \| 42001847 \| 1325 \| 11.97 \| 6.725 \| 17.21 \| 8.327E-06 \| 0.145 \| A/G \| ULK4 \| \| \| 3 \| rs73071261 \| 42003369 \| 1325 \| 11.97 \| 6.725 \| 17.21 \| 8.327E-06 \| 0.145 \| A/C \| ULK4 \| \| \| 3 \| rs73073229 \| 42007850 \| 1325 \| 11.97 \| 6.725 \| 17.21 \| 8.327E-06 \| 0.145 \| C/T \|  \| \| \| 3 \| rs73073234 \| 42012071 \| 1325 \| 11.97 \| 6.725 \| 17.21 \| 8.327E-06 \| 0.145 \| T/C \|  \| \| \| 3 \| rs73073238 \| 42014325 \| 1325 \| 11.97 \| 6.725 \| 17.21 \| 8.327E-06 \| 0.145 \| C/G \|  \| \| \| 3 \| rs73830583 \| 41996948 \| 1325 \| 11.97 \| 6.725 \| 17.21 \| 8.327E-06 \| 0.145 \| T/C \| ULK4 \| \| \| 3 \| rs73830585 \| 42003698 \| 1325 \| 11.97 \| 6.725 \| 17.21 \| 8.327E-06 \| 0.145 \| A/T \|  \| \| \| 3 \| rs78629637 \| 42008781 \| 1325 \| 11.97 \| 6.725 \| 17.21 \| 8.327E-06 \| 0.145 \| T/C \|  \| \| \| 3 \| rs79323474 \| 42000973 \| 1325 \| 11.97 \| 6.725 \| 17.21 \| 8.327E-06 \| 0.145 \| T/C \| ULK4 \| \| \| 17 \| rs112296650 \| 72615967 \| 1325 \| 26.31 \| 14.77 \| 37.85 \| 8.571E-06 \| 0.026 \| G/C \| CD300E \| \| \| 3 \| 3:41953564 \| 41953564 \| 1325 \| 11.91 \| 6.682 \| 17.14 \| 8.656E-06 \| 0.146 \| G/A \| ULK4 \| \| \| 3 \| rs55899232 \| 41959357 \| 1325 \| 11.91 \| 6.682 \| 17.14 \| 8.656E-06 \| 0.146 \| C/T \| ULK4 \| \| \| X \| rs147256925 \| 16305472 \| 1325 \| 47.18 \| 26.47 \| 67.9 \| 8.738E-06 \| 0.012 \| G/T \|  \| \| \| 3 \| rs7639859 \| 42001987 \| 1325 \| 11.91 \| 6.679 \| 17.14 \| 8.753E-06 \| 0.146 \| T/C \| ULK4 \| \| \| 7 \| rs79925923 \| 30885524 \| 1325 \| 14.98 \| 8.402 \| 21.57 \| 8.809E-06 \| 0.091 \| G/T \| INMT-FAM188B \| FAM188B \| \| \| 3 \| rs12108049 \| 41958161 \| 1325 \| 11.89 \| 6.669 \| 17.12 \| 8.811E-06 \| 0.146 \| G/A \| ULK4 \| \| \| 3 \| rs77047799 \| 41958895 \| 1325 \| 11.89 \| 6.669 \| 17.12 \| 8.811E-06 \| 0.146 \| C/T \| ULK4 \| \| \| 3 \| rs113144620 \| 42008312 \| 1325 \| 11.9 \| 6.671 \| 17.13 \| 8.854E-06 \| 0.146 \| G/A \|  \| |  |  |  |  |  |  |  |  |  |  |  |
| --- | --- | --- | --- | --- | --- | --- | --- | --- | --- | --- | --- | --- | --- | --- | --- | --- | --- | --- | --- | --- | --- | --- | --- | --- | --- | --- | --- | --- | --- | --- | --- | --- | --- | --- | --- | --- | --- | --- | --- | --- | --- | --- | --- | --- | --- | --- | --- | --- | --- | --- | --- | --- | --- | --- | --- | --- | --- | --- | --- | --- | --- | --- | --- | --- | --- | --- | --- | --- | --- | --- | --- | --- | --- | --- | --- | --- | --- | --- | --- | --- | --- | --- | --- | --- | --- | --- | --- | --- | --- | --- | --- | --- | --- | --- | --- | --- | --- | --- | --- | --- | --- | --- | --- | --- | --- | --- | --- | --- | --- | --- | --- | --- | --- | --- | --- | --- | --- | --- | --- | --- | --- | --- | --- | --- | --- | --- | --- | --- | --- | --- | --- | --- | --- | --- | --- | --- | --- | --- | --- | --- | --- | --- | --- | --- | --- | --- | --- | --- | --- | --- | --- | --- | --- | --- | --- | --- | --- | --- | --- | --- | --- | --- | --- | --- | --- | --- | --- | --- | --- | --- | --- | --- | --- | --- | --- | --- | --- | --- | --- | --- | --- | --- | --- | --- | --- | --- | --- | --- | --- | --- | --- | --- | --- | --- | --- | --- | --- | --- | --- | --- | --- | --- | --- | --- | --- | --- | --- | --- | --- | --- | --- | --- | --- | --- | --- | --- | --- | --- | --- | --- | --- | --- | --- | --- | --- | --- | --- | --- | --- | --- | --- | --- | --- | --- | --- | --- | --- | --- | --- | --- | --- | --- | --- | --- | --- | --- | --- | --- | --- | --- | --- | --- | --- | --- | --- | --- | --- | --- | --- | --- | --- | --- | --- | --- | --- | --- | --- | --- | --- | --- | --- | --- | --- | --- | --- | --- | --- | --- | --- | --- | --- | --- | --- | --- | --- | --- | --- | --- | --- | --- | --- | --- | --- | --- | --- | --- | --- | --- | --- | --- | --- | --- | --- | --- | --- | --- | --- | --- | --- | --- | --- | --- | --- | --- | --- | --- | --- | --- | --- | --- | --- | --- | --- | --- | --- | --- | --- | --- | --- | --- | --- | --- | --- | --- | --- | --- | --- | --- | --- | --- | --- | --- | --- | --- | --- | --- | --- | --- | --- | --- | --- | --- | --- | --- | --- | --- | --- | --- | --- | --- | --- | --- | --- | --- | --- | --- | --- | --- | --- | --- | --- | --- | --- | --- | --- | --- | --- | --- | --- | --- | --- | --- | --- | --- | --- | --- | --- | --- | --- | --- | --- | --- | --- | --- | --- | --- | --- | --- | --- | --- | --- | --- | --- | --- | --- | --- | --- | --- | --- | --- | --- | --- | --- | --- | --- | --- | --- | --- | --- | --- | --- | --- | --- | --- | --- | --- | --- | --- | --- | --- | --- | --- | --- | --- | --- | --- | --- | --- | --- | --- | --- | --- | --- | --- | --- | --- | --- | --- | --- | --- | --- | --- | --- | --- | --- | --- | --- | --- | --- | --- | --- | --- | --- | --- | --- | --- | --- | --- | --- | --- | --- | --- | --- | --- | --- | --- | --- | --- | --- | --- | --- | --- | --- | --- | --- | --- | --- | --- | --- | --- | --- | --- | --- | --- | --- | --- | --- | --- | --- | --- | --- | --- | --- | --- | --- | --- | --- | --- | --- | --- | --- | --- | --- | --- | --- | --- | --- | --- | --- | --- | --- | --- | --- | --- | --- | --- | --- | --- | --- | --- | --- | --- | --- | --- | --- | --- | --- | --- | --- | --- | --- | --- | --- | --- | --- | --- | --- | --- | --- | --- | --- | --- | --- | --- | --- | --- | --- | --- | --- | --- | --- | --- | --- | --- | --- | --- | --- | --- | --- | --- | --- | --- | --- | --- | --- | --- | --- | --- | --- | --- | --- | --- | --- | --- | --- | --- | --- | --- | --- | --- | --- | --- | --- | --- | --- | --- | --- | --- | --- | --- | --- | --- | --- | --- | --- | --- | --- | --- | --- | --- | --- | --- | --- | --- | --- | --- | --- | --- | --- | --- | --- | --- | --- | --- | --- | --- | --- | --- | --- | --- | --- | --- | --- | --- | --- | --- | --- | --- | --- | --- | --- | --- | --- | --- | --- | --- | --- | --- | --- | --- | --- | --- | --- | --- | --- | --- | --- | --- | --- | --- | --- | --- | --- | --- | --- | --- | --- | --- | --- | --- | --- | --- | --- | --- | --- | --- | --- | --- | --- | --- | --- | --- | --- | --- | --- | --- | --- | --- | --- | --- | --- | --- | --- | --- | --- | --- | --- | --- | --- | --- | --- | --- | --- | --- | --- | --- | --- | --- | --- | --- | --- | --- | --- | --- | --- | --- | --- | --- | --- | --- | --- | --- | --- | --- | --- | --- | --- | --- | --- | --- | --- | --- | --- | --- | --- | --- | --- | --- | --- | --- | --- |

CHR = chromosome; SNP = single nucleotide polymorphism; BP = base pair; L95 = lower 95% confidence interval; U95 = upper 95% confidence interval; MAF = minor allele frequency; GTPS = Guanosine-5′-triphosphates.

### **Supplementary Table 5.** Top 60 variants for the GWAS of apixaban C_min.ss_

| **CHR** | **SNP** | **BP** | **N** | **BETA** | **L95** | **U95** | **P** | **MAF** | **GTPS** | **Gene** |
| --- | --- | --- | --- | --- | --- | --- | --- | --- | --- | --- |
| X | rs147256925 | 16305472 | 1325 | 52.45 | 33.85 | 71.05 | 3.918E-08 | 0.012 | G/T |  |
| 7 | rs78896694 | 14142574 | 1325 | 38.63 | 23.98 | 53.29 | 2.744E-07 | 0.012 | T/C |  |
| 8 | rs143178045 | 14924563 | 1325 | 36.95 | 22.66 | 51.25 | 4.618E-07 | 0.014 | A/C | SGCZ |
| 3 | rs12107681 | 102370270 | 1325 | 12.09 | 7.349 | 16.83 | 6.515E-07 | 0.156 | T/C |  |
| 2 | rs7608316 | 66957093 | 1325 | 34.2 | 20.74 | 47.66 | 7.212E-07 | 0.015 | G/A |  |
| 5 | rs62364239 | 85293185 | 1325 | -18.07 | -25.32 | -10.82 | 1.164E-06 | 0.057 | T/G |  |
| 10 | rs137906597 | 71866622 | 1325 | 28.4 | 16.85 | 39.95 | 1.612E-06 | 0.021 | C/T | H2AFY2 \| AIFM2 |
| 16 | rs59884489 | 49252034 | 1325 | 12.97 | 7.67 | 18.26 | 1.782E-06 | 0.103 | T/C |  |
| X | rs28775972 | 11874988 | 1325 | 35.22 | 20.71 | 49.74 | 2.198E-06 | 0.02 | G/C |  |
| X | rs28778135 | 11872539 | 1325 | 35.22 | 20.71 | 49.74 | 2.198E-06 | 0.02 | G/A |  |
| X | rs5979480 | 11870942 | 1325 | 35.22 | 20.71 | 49.74 | 2.198E-06 | 0.02 | G/C |  |
| X | rs6640800 | 11871811 | 1325 | 35.22 | 20.71 | 49.74 | 2.198E-06 | 0.02 | T/C |  |
| X | rs7888276 | 11868506 | 1325 | 35.22 | 20.71 | 49.74 | 2.198E-06 | 0.02 | G/A |  |
| 3 | 3:42004427 | 42004427 | 1325 | 11.39 | 6.674 | 16.1 | 2.431E-06 | 0.145 | C/A |  |
| 3 | rs113379006 | 42008186 | 1325 | 11.39 | 6.674 | 16.1 | 2.431E-06 | 0.145 | T/C |  |
| 3 | rs114478966 | 42007050 | 1325 | 11.39 | 6.674 | 16.1 | 2.431E-06 | 0.145 | C/T |  |
| 3 | rs55953604 | 42004428 | 1325 | 11.39 | 6.674 | 16.1 | 2.431E-06 | 0.145 | G/A |  |
| 2 | rs56293342 | 162283618 | 1325 | 14.65 | 8.581 | 20.73 | 2.495E-06 | 0.083 | G/C |  |
| 12 | rs138717843 | 123096205 | 1325 | 30.33 | 17.75 | 42.9 | 2.513E-06 | 0.017 | G/A | KNTC1 |
| 3 | rs17284472 | 41996304 | 1325 | 11.25 | 6.54 | 15.96 | 3.125E-06 | 0.146 | A/G | ULK4 |
| 3 | rs6805140 | 41995791 | 1325 | 11.25 | 6.54 | 15.96 | 3.125E-06 | 0.146 | G/A | ULK4 |
| 3 | rs6807562 | 41995655 | 1325 | 11.25 | 6.54 | 15.96 | 3.125E-06 | 0.146 | C/T | ULK4 |
| 3 | 3:42002761 | 42002761 | 1325 | 11.23 | 6.51 | 15.95 | 3.453E-06 | 0.145 | G/A | ULK4 |
| 3 | 3:42008263 | 42008263 | 1325 | 11.23 | 6.51 | 15.95 | 3.453E-06 | 0.145 | A/G |  |
| 3 | rs10510732 | 42007339 | 1325 | 11.23 | 6.51 | 15.95 | 3.453E-06 | 0.145 | A/G |  |
| 3 | rs60966614 | 42005166 | 1325 | 11.23 | 6.51 | 15.95 | 3.453E-06 | 0.145 | G/A |  |
| 3 | rs73071259 | 42001847 | 1325 | 11.23 | 6.51 | 15.95 | 3.453E-06 | 0.145 | A/G | ULK4 |
| 3 | rs73071261 | 42003369 | 1325 | 11.23 | 6.51 | 15.95 | 3.453E-06 | 0.145 | A/C | ULK4 |
| 3 | rs73073229 | 42007850 | 1325 | 11.23 | 6.51 | 15.95 | 3.453E-06 | 0.145 | C/T |  |
| 3 | rs73073234 | 42012071 | 1325 | 11.23 | 6.51 | 15.95 | 3.453E-06 | 0.145 | T/C |  |
| 3 | rs73073238 | 42014325 | 1325 | 11.23 | 6.51 | 15.95 | 3.453E-06 | 0.145 | C/G |  |
| 3 | rs73830583 | 41996948 | 1325 | 11.23 | 6.51 | 15.95 | 3.453E-06 | 0.145 | T/C | ULK4 |
| 3 | rs73830585 | 42003698 | 1325 | 11.23 | 6.51 | 15.95 | 3.453E-06 | 0.145 | A/T |  |
| 3 | rs78629637 | 42008781 | 1325 | 11.23 | 6.51 | 15.95 | 3.453E-06 | 0.145 | T/C |  |
| 3 | rs79323474 | 42000973 | 1325 | 11.23 | 6.51 | 15.95 | 3.453E-06 | 0.145 | T/C | ULK4 |
| X | rs143083304 | 16422848 | 1325 | 36.66 | 21.21 | 52.11 | 3.636E-06 | 0.016 | T/C |  |
| 3 | rs113144620 | 42008312 | 1325 | 11.15 | 6.44 | 15.86 | 0.00000383 | 0.146 | G/A |  |
| 3 | rs73073226 | 42005838 | 1325 | 11.15 | 6.44 | 15.86 | 0.00000383 | 0.146 | C/T |  |
| 3 | rs74947509 | 42006323 | 1325 | 11.15 | 6.44 | 15.86 | 0.00000383 | 0.146 | C/T |  |
| 3 | rs7639859 | 42001987 | 1325 | 11.15 | 6.441 | 15.86 | 3.833E-06 | 0.146 | T/C | ULK4 |
| 4 | rs151244651 | 178345957 | 1325 | 29.77 | 17.19 | 42.36 | 3.887E-06 | 0.018 | A/G |  |
| 12 | rs117943690 | 76590246 | 1325 | 28.88 | 16.65 | 41.11 | 4.071E-06 | 0.018 | T/C |  |
| 3 | rs12108049 | 41958161 | 1325 | 11.1 | 6.395 | 15.81 | 4.155E-06 | 0.146 | G/A | ULK4 |
| 3 | rs77047799 | 41958895 | 1325 | 11.1 | 6.395 | 15.81 | 4.155E-06 | 0.146 | C/T | ULK4 |
| 3 | rs73071238 | 41992243 | 1325 | 11.11 | 6.402 | 15.82 | 4.157E-06 | 0.146 | G/A | ULK4 |
| 3 | 3:41953564 | 41953564 | 1325 | 11.1 | 6.386 | 15.8 | 4.251E-06 | 0.146 | G/A | ULK4 |
| 3 | rs55899232 | 41959357 | 1325 | 11.1 | 6.386 | 15.8 | 4.251E-06 | 0.146 | C/T | ULK4 |
| 3 | rs111419796 | 41976968 | 1325 | 11.09 | 6.377 | 15.8 | 4.351E-06 | 0.146 | A/G | ULK4 |
| 3 | rs11129935 | 41985203 | 1325 | 11.08 | 6.369 | 15.8 | 4.462E-06 | 0.146 | C/A | ULK4 |
| 3 | rs112787664 | 41992441 | 1325 | 11.08 | 6.369 | 15.8 | 4.462E-06 | 0.146 | T/C | ULK4 |
| 3 | rs113300894 | 41984243 | 1325 | 11.08 | 6.369 | 15.8 | 4.462E-06 | 0.146 | C/G | ULK4 |
| 3 | rs113803925 | 41989859 | 1325 | 11.08 | 6.369 | 15.8 | 4.462E-06 | 0.146 | T/C | ULK4 |
| 3 | rs113923306 | 41994948 | 1325 | 11.08 | 6.369 | 15.8 | 4.462E-06 | 0.146 | T/C | ULK4 |
| 3 | rs138732282 | 41971960 | 1325 | 11.08 | 6.369 | 15.8 | 4.462E-06 | 0.146 | A/C | ULK4 |
| 3 | rs140020439 | 41990978 | 1325 | 11.08 | 6.369 | 15.8 | 4.462E-06 | 0.146 | A/G | ULK4 |
| 3 | rs142839172 | 41994984 | 1325 | 11.08 | 6.369 | 15.8 | 4.462E-06 | 0.146 | A/G | ULK4 |
| 3 | rs142874902 | 41991986 | 1325 | 11.08 | 6.369 | 15.8 | 4.462E-06 | 0.146 | T/C | ULK4 |
| 3 | rs150394401 | 41970230 | 1325 | 11.08 | 6.369 | 15.8 | 4.462E-06 | 0.146 | T/G | ULK4 |
| 3 | rs17218264 | 41975847 | 1325 | 11.08 | 6.369 | 15.8 | 4.462E-06 | 0.146 | C/T | ULK4 |
| 3 | rs17218441 | 41987044 | 1325 | 11.08 | 6.369 | 15.8 | 4.462E-06 | 0.146 | G/A | ULK4 |

CHR = chromosome; SNP = single nucleotide polymorphism; BP = base pair; L95 = lower 95% confidence interval; U95 = upper 95% confidence interval; MAF = minor allele frequency; GTPS = Guanosine-5′-triphosphates.

### **Supplementary Table 6.** Linear regression result for rs2231142 versus apixaban PK parameters adjusted for clinical factors

| **SNP** | **Variable** | **N** | **Beta** | **CI** | **P** | **R^2^** | **Variable R^2^** | **Partial R^2^** |
| --- | --- | --- | --- | --- | --- | --- | --- | --- |
| rs2231142 | AUC_ss_ | 1325 | 195.327 | (102.38-288.274) | 4.04e-05 | 0.464 | 0.009 | 0.007 |
| rs2231142 | C_max,ss_ | 1325 | 11.707 | (5.982-17.432) | 6.47e-05 | 0.315 | 0.011 | 0.008 |
| rs2231142 | C_min,ss_ | 1325 | 10.883 | (5.767- 16) | 3.26e-05 | 0.262 | 0.011 | 0.010 |

The clinical factors included in the model are age, gender, calculated creatinine clearance (CrCl) at baseline (ml/min), weight (kg), dose of apixaban, and concomitant treatment with amiodarone or calcium channel blockers. Note that this model does not include the six genetic principal components, hence the estimates differ from the GWAS results.

R^2^ = amount of variance explained by the model, Variable R^2^ = amount of variance explained by the variable on its own, Partial R^2^ = the increase in amount of variance explained if the variable is added last to a model including all other variables.

### **Supplementary Table 7.** Top ten SNPs per apixaban PK parameter in the candidate gene analyses, adjusted for the covariates listed in Table 3.

|  | **CHR** | **SNP** | **BP** | **N** | **BETA** | **L95** | **U95** | **P** | **MAF** | **GTPS** | **Gene** |
| --- | --- | --- | --- | --- | --- | --- | --- | --- | --- | --- | --- |
| **AUC_ss_** | 4 | rs3114018 | 89064581 | 1325 | -106.8 | -166.2 | -47.42 | 0.0004371 | 0.466 | A/C | ABCG2 |
|  | 4 | rs1481012 | 89039082 | 1325 | 152.7 | 60.65 | 244.7 | 0.001178 | 0.116 | G/A | ABCG2 |
|  | 4 | rs138409370 | 89044312 | 1325 | 151 | 59.15 | 242.8 | 0.001303 | 0.118 | T/A | ABCG2 |
|  | 4 | rs141471965 | 89046202 | 1325 | 151 | 59.15 | 242.8 | 0.001303 | 0.118 | T/C | ABCG2 |
|  | 4 | rs149027545 | 89044180 | 1325 | 151 | 59.15 | 242.8 | 0.001303 | 0.118 | C/G | ABCG2 |
|  | 4 | rs2231142 | 89052323 | 1325 | 151 | 59.15 | 242.8 | 0.001303 | 0.118 | T/G | ABCG2 |
|  | 4 | rs45499402 | 89043634 | 1325 | 151 | 59.15 | 242.8 | 0.001303 | 0.118 | C/G | ABCG2 |
|  | 4 | rs74904971 | 89050026 | 1325 | 151 | 59.15 | 242.8 | 0.001303 | 0.118 | A/C | ABCG2 |
|  | 4 | rs2199936 | 89045331 | 1325 | 146.6 | 55.67 | 237.5 | 0.001611 | 0.12 | A/G | ABCG2 |
|  | 4 | rs4148155 | 89054667 | 1325 | 146.9 | 55.12 | 238.6 | 0.001743 | 0.118 | G/A | ABCG2 |
| **C_max,ss_** | 4 | rs3114018 | 89064581 | 1325 | -6.589 | -10.27 | -2.904 | 0.0004734 | 0.466 | A/C | ABCG2 |
|  | 4 | rs138409370 | 89044312 | 1325 | 9.633 | 3.934 | 15.33 | 0.0009494 | 0.118 | T/A | ABCG2 |
|  | 4 | rs141471965 | 89046202 | 1325 | 9.633 | 3.934 | 15.33 | 0.0009494 | 0.118 | T/C | ABCG2 |
|  | 4 | rs149027545 | 89044180 | 1325 | 9.633 | 3.934 | 15.33 | 0.0009494 | 0.118 | C/G | ABCG2 |
|  | 4 | rs2231142 | 89052323 | 1325 | 9.633 | 3.934 | 15.33 | 0.0009494 | 0.118 | T/G | ABCG2 |
|  | 4 | rs45499402 | 89043634 | 1325 | 9.633 | 3.934 | 15.33 | 0.0009494 | 0.118 | C/G | ABCG2 |
|  | 4 | rs74904971 | 89050026 | 1325 | 9.633 | 3.934 | 15.33 | 0.0009494 | 0.118 | A/C | ABCG2 |
|  | 4 | rs4148155 | 89054667 | 1325 | 9.477 | 3.783 | 15.17 | 0.001135 | 0.118 | G/A | ABCG2 |
|  | 4 | rs1481012 | 89039082 | 1325 | 9.303 | 3.588 | 15.02 | 0.001454 | 0.116 | G/A | ABCG2 |
|  | 4 | rs2199936 | 89045331 | 1325 | 9.001 | 3.358 | 14.64 | 0.001808 | 0.12 | A/G | ABCG2 |
| **C_min,ss_** | 4 | rs138409370 | 89044312 | 1325 | 9.695 | 4.562 | 14.83 | 0.0002226 | 0.118 | T/A | ABCG2 |
|  | 4 | rs141471965 | 89046202 | 1325 | 9.695 | 4.562 | 14.83 | 0.0002226 | 0.118 | T/C | ABCG2 |
|  | 4 | rs149027545 | 89044180 | 1325 | 9.695 | 4.562 | 14.83 | 0.0002226 | 0.118 | C/G | ABCG2 |
|  | 4 | rs2231142 | 89052323 | 1325 | 9.695 | 4.562 | 14.83 | 0.0002226 | 0.118 | T/G | ABCG2 |
|  | 4 | rs45499402 | 89043634 | 1325 | 9.695 | 4.562 | 14.83 | 0.0002226 | 0.118 | C/G | ABCG2 |
|  | 4 | rs74904971 | 89050026 | 1325 | 9.695 | 4.562 | 14.83 | 0.0002226 | 0.118 | A/C | ABCG2 |
|  | 4 | rs4148155 | 89054667 | 1325 | 9.527 | 4.399 | 14.65 | 0.0002818 | 0.118 | G/A | ABCG2 |
|  | 4 | rs1481012 | 89039082 | 1325 | 9.442 | 4.296 | 14.59 | 0.0003351 | 0.116 | G/A | ABCG2 |
|  | 4 | rs3114018 | 89064581 | 1325 | -6.061 | -9.383 | -2.739 | 0.000361 | 0.466 | A/C | ABCG2 |
|  | 4 | rs2199936 | 89045331 | 1325 | 9.171 | 4.09 | 14.25 | 0.0004181 | 0.12 | A/G | ABCG2 |

CHR = chromosome; SNP = single nucleotide polymorphism; BP = base pair; L95 = lower 95% confidence interval; U95 = upper 95% confidence interval; MAF = minor allele frequency; GTPS = Genotypes minor/major allele; AUCss = area under the curve at steady state; Cmax ss = maximum serum concentration at steady state; Cmin ss = serum trough concentration at steady state.

### **Supplementary Table 8.** Top 60 variants for the candidate gene analyses of apixaban AUC_ss_

| **CHR** | **SNP** | **BP** | **N** | **BETA** | **L95** | **U95** | **P** | **MAF** | **GTPS** | **Gene** |
| --- | --- | --- | --- | --- | --- | --- | --- | --- | --- | --- |
| 4 | rs3114018 | 89064581 | 1325 | -106.8 | -166.2 | -47.42 | 0.0004371 | 0.466 | A/C | ABCG2 |
| 4 | rs1481012 | 89039082 | 1325 | 152.7 | 60.65 | 244.7 | 0.001178 | 0.116 | G/A | ABCG2 |
| 4 | rs138409370 | 89044312 | 1325 | 151 | 59.15 | 242.8 | 0.001303 | 0.118 | T/A | ABCG2 |
| 4 | rs141471965 | 89046202 | 1325 | 151 | 59.15 | 242.8 | 0.001303 | 0.118 | T/C | ABCG2 |
| 4 | rs149027545 | 89044180 | 1325 | 151 | 59.15 | 242.8 | 0.001303 | 0.118 | C/G | ABCG2 |
| 4 | rs2231142 | 89052323 | 1325 | 151 | 59.15 | 242.8 | 0.001303 | 0.118 | T/G | ABCG2 |
| 4 | rs45499402 | 89043634 | 1325 | 151 | 59.15 | 242.8 | 0.001303 | 0.118 | C/G | ABCG2 |
| 4 | rs74904971 | 89050026 | 1325 | 151 | 59.15 | 242.8 | 0.001303 | 0.118 | A/C | ABCG2 |
| 4 | rs2199936 | 89045331 | 1325 | 146.6 | 55.67 | 237.5 | 0.001611 | 0.12 | A/G | ABCG2 |
| 4 | rs4148155 | 89054667 | 1325 | 146.9 | 55.12 | 238.6 | 0.001743 | 0.118 | G/A | ABCG2 |
| 4 | rs2728125 | 89001893 | 1325 | 148.7 | 55.44 | 242 | 0.001816 | 0.11 | G/A |  |
| 4 | rs2231156 | 89020427 | 1325 | 147.3 | 54.8 | 239.9 | 0.001843 | 0.109 | A/C | ABCG2 |
| 4 | rs2054576 | 89028775 | 1325 | 147.1 | 54.67 | 239.6 | 0.001856 | 0.109 | G/A | ABCG2 |
| 4 | rs4148157 | 89020934 | 1325 | 147.1 | 54.67 | 239.6 | 0.001856 | 0.109 | A/G | ABCG2 |
| 4 | rs4693924 | 89023224 | 1325 | 147.1 | 54.67 | 239.6 | 0.001856 | 0.109 | A/G | ABCG2 |
| 4 | rs1383585 | 89019735 | 1325 | 145.9 | 53.44 | 238.3 | 0.002022 | 0.109 | G/A | ABCG2 |
| 4 | rs3109823 | 89064602 | 1325 | -106.2 | -174.2 | -38.32 | 0.002215 | 0.271 | C/T | ABCG2 |
| 4 | rs76979899 | 89025241 | 1325 | 143.6 | 50.68 | 236.5 | 0.002503 | 0.109 | T/C | ABCG2 |
| 7 | rs28381779 | 87246983 | 1325 | 122.4 | 39.37 | 205.5 | 0.003925 | 0.142 | A/G | ABCB1 |
| 4 | rs3109822 | 89075223 | 1325 | -105.6 | -177.3 | -33.88 | 0.00397 | 0.228 | T/C | ABCG2 |
| 4 | rs6821607 | 89074808 | 1325 | -105.2 | -177 | -33.52 | 0.004093 | 0.227 | A/G | ABCG2 |
| 7 | rs74491457 | 87348996 | 1325 | 120.9 | 37.45 | 204.4 | 0.004592 | 0.143 | G/A | RUNDC3B |
| 4 | rs2622604 | 89078924 | 1325 | -103.4 | -175.1 | -31.67 | 0.004794 | 0.228 | T/C | ABCG2 |
| 7 | rs28381724 | 87340535 | 1325 | 119.2 | 36.15 | 202.2 | 0.004979 | 0.142 | A/G | ABCB1 \| RUNDC3B |
| 7 | rs75232677 | 87206908 | 1325 | 116.6 | 34.5 | 198.6 | 0.005448 | 0.147 | C/T | ABCB1 |
| 7 | rs28381784 | 87238517 | 1325 | 218.6 | 63.48 | 373.7 | 0.005821 | 0.038 | G/A | ABCB1 |
| 4 | rs3114019 | 89081441 | 1325 | -99.92 | -171.8 | -28 | 0.006556 | 0.227 | C/T | ABCG2 |
| 7 | rs28746490 | 87297189 | 1325 | -282.4 | -486.1 | -78.73 | 0.006666 | 0.021 | T/C | ABCB1 \| RUNDC3B |
| 4 | rs2622606 | 89084381 | 1325 | -99.13 | -171 | -27.23 | 0.006978 | 0.228 | A/T | ABCG2 |
| 7 | rs74569377 | 87208381 | 1325 | 111.6 | 29.6 | 193.7 | 0.007746 | 0.147 | C/T | ABCB1 |
| 4 | rs17788087 | 89056565 | 1325 | 273.7 | 71.85 | 475.6 | 0.007966 | 0.021 | C/A | ABCG2 |
| 16 | rs4787453 | 28609963 | 1325 | 81.02 | 20.78 | 141.3 | 0.00849 | 0.48 | C/G | NPIPL1 |
| 4 | rs142578439 | 89056973 | 1325 | 240 | 60.24 | 419.9 | 0.008984 | 0.028 | G/T | ABCG2 |
| 7 | 7:87172053 | 87172053 | 1325 | -166 | -291.6 | -40.46 | 0.009661 | 0.05 | A/G | ABCB1 |
| 4 | rs2622620 | 89063851 | 1325 | 78.24 | 18.4 | 138.1 | 0.01051 | 0.478 | A/C | ABCG2 |
| 7 | rs4148733 | 87213232 | 1325 | 103.7 | 24.34 | 183 | 0.01054 | 0.161 | G/A | ABCB1 |
| 7 | rs73387255 | 87211486 | 1325 | 103.7 | 24.34 | 183 | 0.01054 | 0.161 | A/G | ABCB1 |
| 7 | rs4148732 | 87234049 | 1325 | 108.1 | 25.36 | 190.8 | 0.01055 | 0.144 | C/T | ABCB1 |
| 7 | rs147690884 | 87187189 | 1325 | 209.4 | 48.36 | 370.5 | 0.01093 | 0.035 | A/T | ABCB1 |
| 7 | rs2229109 | 87179809 | 1325 | 205.4 | 45.84 | 365 | 0.01175 | 0.035 | T/C | ABCB1 |
| 7 | rs17327442 | 87212990 | 1325 | 100.9 | 21.75 | 180 | 0.01259 | 0.163 | A/T | ABCB1 |
| 7 | rs117937072 | 87164349 | 1325 | 205.9 | 44.02 | 367.8 | 0.0128 | 0.034 | C/A | ABCB1 |
| 7 | rs150409141 | 87206955 | 1325 | 199.4 | 39.01 | 359.7 | 0.01495 | 0.035 | T/C | ABCB1 |
| 7 | rs117773249 | 87212404 | 1325 | 195.6 | 36.7 | 354.4 | 0.01597 | 0.036 | A/G | ABCB1 |
| 7 | rs181465270 | 87351586 | 1325 | 292.5 | 51.1 | 533.8 | 0.0177 | 0.015 | C/T | RUNDC3B |
| 7 | rs150515188 | 87346068 | 1325 | -241.3 | -441.9 | -40.79 | 0.01849 | 0.022 | T/C | RUNDC3B |
| 7 | rs17327624 | 87216817 | 1325 | 87.73 | 14.52 | 160.9 | 0.01899 | 0.208 | T/G | ABCB1 |
| 4 | 4:89066715 | 89066715 | 1325 | -70.81 | -130.2 | -11.43 | 0.01957 | 0.462 | C/A | ABCG2 |
| 7 | rs56227693 | 87200734 | 1325 | 265.8 | 39.52 | 492.1 | 0.02148 | 0.018 | C/G | ABCB1 |
| 4 | rs17731799 | 89068455 | 1325 | 70.21 | 10.17 | 130.3 | 0.02206 | 0.469 | T/G | ABCG2 |
| 4 | rs2622627 | 89065353 | 1325 | -69.09 | -128.5 | -9.637 | 0.02291 | 0.463 | C/A | ABCG2 |
| 4 | rs2725249 | 89065868 | 1325 | -69.09 | -128.5 | -9.637 | 0.02291 | 0.463 | A/C | ABCG2 |
| 4 | rs2725246 | 89068498 | 1325 | 69.34 | 9.247 | 129.4 | 0.02389 | 0.47 | A/G | ABCG2 |
| 4 | rs2622624 | 89069406 | 1325 | 69.39 | 9.178 | 129.6 | 0.02406 | 0.471 | C/T | ABCG2 |
| 4 | rs2725245 | 89068738 | 1325 | 68.66 | 8.524 | 128.8 | 0.0254 | 0.47 | A/G | ABCG2 |
| 4 | rs2725242 | 89069527 | 1325 | -67.16 | -126.5 | -7.789 | 0.02679 | 0.463 | T/A | ABCG2 |
| 7 | rs28381834 | 87200332 | 1325 | 251.8 | 27.79 | 475.8 | 0.02776 | 0.018 | T/G | ABCB1 |
| 7 | rs118078935 | 87189473 | 1325 | 256.8 | 28.02 | 485.6 | 0.02798 | 0.017 | C/T | ABCB1 |
| 4 | rs2725244 | 89068761 | 1325 | -66.42 | -125.8 | -7.043 | 0.02852 | 0.463 | T/C | ABCG2 |
| 4 | rs2725247 | 89068257 | 1325 | 66.84 | 6.527 | 127.2 | 0.03003 | 0.472 | A/G | ABCG2 |

CHR = chromosome; SNP = single nucleotide polymorphism; BP = base pair; L95 = lower 95% confidence interval; U95 = upper 95% confidence interval; MAF = minor allele frequency; GTPS = Guanosine-5′-triphosphates.

### **Supplementary Table 9.** Top 60 variants for the candidate gene analyses of apixaban C_max.ss_

| **CHR** | **SNP** | **BP** | **N** | **BETA** | **L95** | **U95** | **P** | **MAF** | **GTPS** | **Gene** |
| --- | --- | --- | --- | --- | --- | --- | --- | --- | --- | --- |
| 4 | rs3114018 | 89064581 | 1325 | -6.589 | -10.27 | -2.904 | 0.0004734 | 0.466 | A/C | ABCG2 |
| 4 | rs138409370 | 89044312 | 1325 | 9.633 | 3.934 | 15.33 | 0.0009494 | 0.118 | T/A | ABCG2 |
| 4 | rs141471965 | 89046202 | 1325 | 9.633 | 3.934 | 15.33 | 0.0009494 | 0.118 | T/C | ABCG2 |
| 4 | rs149027545 | 89044180 | 1325 | 9.633 | 3.934 | 15.33 | 0.0009494 | 0.118 | C/G | ABCG2 |
| 4 | rs2231142 | 89052323 | 1325 | 9.633 | 3.934 | 15.33 | 0.0009494 | 0.118 | T/G | ABCG2 |
| 4 | rs45499402 | 89043634 | 1325 | 9.633 | 3.934 | 15.33 | 0.0009494 | 0.118 | C/G | ABCG2 |
| 4 | rs74904971 | 89050026 | 1325 | 9.633 | 3.934 | 15.33 | 0.0009494 | 0.118 | A/C | ABCG2 |
| 4 | rs4148155 | 89054667 | 1325 | 9.477 | 3.783 | 15.17 | 0.001135 | 0.118 | G/A | ABCG2 |
| 4 | rs1481012 | 89039082 | 1325 | 9.303 | 3.588 | 15.02 | 0.001454 | 0.116 | G/A | ABCG2 |
| 4 | rs2199936 | 89045331 | 1325 | 9.001 | 3.358 | 14.64 | 0.001808 | 0.12 | A/G | ABCG2 |
| 4 | rs17788087 | 89056565 | 1325 | 19.63 | 7.112 | 32.15 | 0.002162 | 0.021 | C/A | ABCG2 |
| 7 | rs75232677 | 87206908 | 1325 | 7.933 | 2.841 | 13.02 | 0.002307 | 0.147 | C/T | ABCB1 |
| 4 | rs3109823 | 89064602 | 1325 | -6.567 | -10.78 | -2.351 | 0.002313 | 0.271 | C/T | ABCG2 |
| 7 | rs56227693 | 87200734 | 1325 | 21.26 | 7.236 | 35.29 | 0.003024 | 0.018 | C/G | ABCB1 |
| 7 | rs74569377 | 87208381 | 1325 | 7.71 | 2.62 | 12.8 | 0.003043 | 0.147 | C/T | ABCB1 |
| 4 | rs2231156 | 89020427 | 1325 | 8.688 | 2.942 | 14.43 | 0.003099 | 0.109 | A/C | ABCG2 |
| 4 | rs1383585 | 89019735 | 1325 | 8.672 | 2.933 | 14.41 | 0.003117 | 0.109 | G/A | ABCG2 |
| 4 | rs2054576 | 89028775 | 1325 | 8.622 | 2.88 | 14.36 | 0.00331 | 0.109 | G/A | ABCG2 |
| 4 | rs4148157 | 89020934 | 1325 | 8.622 | 2.88 | 14.36 | 0.00331 | 0.109 | A/G | ABCG2 |
| 4 | rs4693924 | 89023224 | 1325 | 8.622 | 2.88 | 14.36 | 0.00331 | 0.109 | A/G | ABCG2 |
| 4 | rs3109822 | 89075223 | 1325 | -6.677 | -11.13 | -2.224 | 0.003349 | 0.228 | T/C | ABCG2 |
| 4 | rs6821607 | 89074808 | 1325 | -6.639 | -11.09 | -2.187 | 0.003528 | 0.227 | A/G | ABCG2 |
| 7 | rs28381779 | 87246983 | 1325 | 7.645 | 2.49 | 12.8 | 0.003715 | 0.142 | A/G | ABCB1 |
| 7 | rs28381834 | 87200332 | 1325 | 20.55 | 6.664 | 34.44 | 0.003788 | 0.018 | T/G | ABCB1 |
| 4 | rs2728125 | 89001893 | 1325 | 8.53 | 2.737 | 14.32 | 0.003966 | 0.11 | G/A |  |
| 4 | rs2622604 | 89078924 | 1325 | -6.543 | -11 | -2.091 | 0.004036 | 0.228 | T/C | ABCG2 |
| 7 | rs181465270 | 87351586 | 1325 | 21.85 | 6.881 | 36.82 | 0.004292 | 0.015 | C/T | RUNDC3B |
| 4 | rs3114019 | 89081441 | 1325 | -6.433 | -10.9 | -1.969 | 0.00481 | 0.227 | C/T | ABCG2 |
| 4 | rs76979899 | 89025241 | 1325 | 8.282 | 2.511 | 14.05 | 0.004984 | 0.109 | T/C | ABCG2 |
| 4 | rs2622606 | 89084381 | 1325 | -6.393 | -10.86 | -1.93 | 0.005062 | 0.228 | A/T | ABCG2 |
| 7 | rs4148733 | 87213232 | 1325 | 7.024 | 2.1 | 11.95 | 0.005248 | 0.161 | G/A | ABCB1 |
| 7 | rs73387255 | 87211486 | 1325 | 7.024 | 2.1 | 11.95 | 0.005248 | 0.161 | A/G | ABCB1 |
| 7 | 7:87137015 | 87137015 | 1325 | 5.856 | 1.743 | 9.969 | 0.005335 | 0.262 | T/C | ABCB1 |
| 7 | rs6979885 | 87137461 | 1325 | 5.856 | 1.743 | 9.969 | 0.005335 | 0.262 | A/G | ABCB1 |
| 4 | rs2622620 | 89063851 | 1325 | 5.274 | 1.561 | 8.988 | 0.005451 | 0.478 | A/C | ABCG2 |
| 7 | rs118078935 | 87189473 | 1325 | 20.12 | 5.936 | 34.31 | 0.005515 | 0.017 | C/T | ABCB1 |
| 7 | rs17327624 | 87216817 | 1325 | 6.352 | 1.811 | 10.89 | 0.006202 | 0.208 | T/G | ABCB1 |
| 7 | 7:87172053 | 87172053 | 1325 | -10.85 | -18.65 | -3.063 | 0.006411 | 0.05 | A/G | ABCB1 |
| 7 | rs28381724 | 87340535 | 1325 | 7.133 | 1.977 | 12.29 | 0.006785 | 0.142 | A/G | ABCB1 \| RUNDC3B |
| 7 | rs56126905 | 87157049 | 1325 | 19.83 | 5.476 | 34.19 | 0.006864 | 0.017 | C/T | ABCB1 |
| 7 | rs74491457 | 87348996 | 1325 | 7.149 | 1.966 | 12.33 | 0.006952 | 0.143 | G/A | RUNDC3B |
| 4 | 4:89066715 | 89066715 | 1325 | -5.061 | -8.744 | -1.377 | 0.007176 | 0.462 | C/A | ABCG2 |
| 7 | rs28746490 | 87297189 | 1325 | -17.2 | -29.85 | -4.551 | 0.007789 | 0.021 | T/C | ABCB1 \| RUNDC3B |
| 7 | rs17327442 | 87212990 | 1325 | 6.615 | 1.706 | 11.52 | 0.008368 | 0.163 | A/T | ABCB1 |
| 4 | rs17731799 | 89068455 | 1325 | 4.971 | 1.246 | 8.696 | 0.009006 | 0.469 | T/G | ABCG2 |
| 4 | rs2622627 | 89065353 | 1325 | -4.9 | -8.588 | -1.211 | 0.009326 | 0.463 | C/A | ABCG2 |
| 4 | rs2725249 | 89065868 | 1325 | -4.9 | -8.588 | -1.211 | 0.009326 | 0.463 | A/C | ABCG2 |
| 4 | rs2725246 | 89068498 | 1325 | 4.948 | 1.22 | 8.676 | 0.009392 | 0.47 | A/G | ABCG2 |
| 4 | rs2725245 | 89068738 | 1325 | 4.93 | 1.2 | 8.661 | 0.009697 | 0.47 | A/G | ABCG2 |
| 4 | rs2622624 | 89069406 | 1325 | 4.917 | 1.182 | 8.653 | 0.009983 | 0.471 | C/T | ABCG2 |
| 4 | rs2725244 | 89068761 | 1325 | -4.844 | -8.528 | -1.161 | 0.01006 | 0.463 | T/C | ABCG2 |
| 4 | rs2725242 | 89069527 | 1325 | -4.837 | -8.52 | -1.153 | 0.01017 | 0.463 | T/A | ABCG2 |
| 7 | rs4148732 | 87234049 | 1325 | 6.682 | 1.548 | 11.82 | 0.01086 | 0.144 | C/T | ABCB1 |
| 7 | rs4148747 | 87147147 | 1325 | -8.03 | -14.24 | -1.821 | 0.01136 | 0.1 | A/T | ABCB1 |
| 7 | rs28401781 | 87148328 | 1325 | -8.132 | -14.44 | -1.821 | 0.01167 | 0.098 | T/C | ABCB1 |
| 4 | rs2725247 | 89068257 | 1325 | 4.82 | 1.078 | 8.562 | 0.01169 | 0.472 | A/G | ABCG2 |
| 7 | rs1882478 | 87137018 | 1325 | 5.162 | 1.152 | 9.172 | 0.01175 | 0.311 | T/C | ABCB1 |
| 7 | rs150515188 | 87346068 | 1325 | -15.84 | -28.29 | -3.393 | 0.01274 | 0.022 | T/C | RUNDC3B |
| 7 | rs28381829 | 87213878 | 1325 | 20.37 | 4.363 | 36.37 | 0.01275 | 0.014 | A/G | ABCB1 |
| 4 | rs34633905 | 89074771 | 1325 | 4.785 | 1.022 | 8.548 | 0.01281 | 0.471 | G/C | ABCG2 |

CHR = chromosome; SNP = single nucleotide polymorphism; BP = base pair; L95 = lower 95% confidence interval; U95 = upper 95% confidence interval; MAF = minor allele frequency; GTPS = Guanosine-5′-triphosphates.

### **Supplementary Table 10.** Top 60 variants for the candidate gene analyses of apixaban C_min.ss_

| **CHR** | **SNP** | **BP** | **N** | **BETA** | **L95** | **U95** | **P** | **MAF** | **GTPS** | **Gene** |
| --- | --- | --- | --- | --- | --- | --- | --- | --- | --- | --- |
| 4 | rs138409370 | 89044312 | 1325 | 9.695 | 4.562 | 14.83 | 0.0002226 | 0.118 | T/A | ABCG2 |
| 4 | rs141471965 | 89046202 | 1325 | 9.695 | 4.562 | 14.83 | 0.0002226 | 0.118 | T/C | ABCG2 |
| 4 | rs149027545 | 89044180 | 1325 | 9.695 | 4.562 | 14.83 | 0.0002226 | 0.118 | C/G | ABCG2 |
| 4 | rs2231142 | 89052323 | 1325 | 9.695 | 4.562 | 14.83 | 0.0002226 | 0.118 | T/G | ABCG2 |
| 4 | rs45499402 | 89043634 | 1325 | 9.695 | 4.562 | 14.83 | 0.0002226 | 0.118 | C/G | ABCG2 |
| 4 | rs74904971 | 89050026 | 1325 | 9.695 | 4.562 | 14.83 | 0.0002226 | 0.118 | A/C | ABCG2 |
| 4 | rs4148155 | 89054667 | 1325 | 9.527 | 4.399 | 14.65 | 0.0002818 | 0.118 | G/A | ABCG2 |
| 4 | rs1481012 | 89039082 | 1325 | 9.442 | 4.296 | 14.59 | 0.0003351 | 0.116 | G/A | ABCG2 |
| 4 | rs3114018 | 89064581 | 1325 | -6.061 | -9.383 | -2.739 | 0.000361 | 0.466 | A/C | ABCG2 |
| 4 | rs2199936 | 89045331 | 1325 | 9.171 | 4.09 | 14.25 | 0.0004181 | 0.12 | A/G | ABCG2 |
| 4 | rs2231156 | 89020427 | 1325 | 9.209 | 4.036 | 14.38 | 0.0005009 | 0.109 | A/C | ABCG2 |
| 4 | rs1383585 | 89019735 | 1325 | 9.189 | 4.022 | 14.36 | 0.000507 | 0.109 | G/A | ABCG2 |
| 4 | rs2054576 | 89028775 | 1325 | 9.164 | 3.994 | 14.33 | 0.0005291 | 0.109 | G/A | ABCG2 |
| 4 | rs4148157 | 89020934 | 1325 | 9.164 | 3.994 | 14.33 | 0.0005291 | 0.109 | A/G | ABCG2 |
| 4 | rs4693924 | 89023224 | 1325 | 9.164 | 3.994 | 14.33 | 0.0005291 | 0.109 | A/G | ABCG2 |
| 4 | rs2728125 | 89001893 | 1325 | 8.817 | 3.6 | 14.03 | 0.0009491 | 0.11 | G/A |  |
| 4 | rs76979899 | 89025241 | 1325 | 8.773 | 3.577 | 13.97 | 0.0009613 | 0.109 | T/C | ABCG2 |
| 7 | rs75232677 | 87206908 | 1325 | 7.629 | 3.041 | 12.22 | 0.001146 | 0.147 | C/T | ABCB1 |
| 4 | rs17788087 | 89056565 | 1325 | 18.34 | 7.061 | 29.63 | 0.001475 | 0.021 | C/A | ABCG2 |
| 7 | rs74569377 | 87208381 | 1325 | 7.444 | 2.858 | 12.03 | 0.001499 | 0.147 | C/T | ABCB1 |
| 7 | rs28381779 | 87246983 | 1325 | 7.421 | 2.776 | 12.07 | 0.001777 | 0.142 | A/G | ABCB1 |
| 4 | rs3109823 | 89064602 | 1325 | -5.991 | -9.791 | -2.19 | 0.002047 | 0.271 | C/T | ABCG2 |
| 4 | rs3109822 | 89075223 | 1325 | -6.312 | -10.32 | -2.3 | 0.002089 | 0.228 | T/C | ABCG2 |
| 7 | rs4148733 | 87213232 | 1325 | 6.938 | 2.503 | 11.37 | 0.002214 | 0.161 | G/A | ABCB1 |
| 7 | rs73387255 | 87211486 | 1325 | 6.938 | 2.503 | 11.37 | 0.002214 | 0.161 | A/G | ABCB1 |
| 4 | rs6821607 | 89074808 | 1325 | -6.274 | -10.29 | -2.263 | 0.002219 | 0.227 | A/G | ABCG2 |
| 4 | rs2622604 | 89078924 | 1325 | -6.201 | -10.21 | -2.189 | 0.002498 | 0.228 | T/C | ABCG2 |
| 4 | rs3114019 | 89081441 | 1325 | -6.133 | -10.16 | -2.111 | 0.002855 | 0.227 | C/T | ABCG2 |
| 4 | rs2622606 | 89084381 | 1325 | -6.11 | -10.13 | -2.088 | 0.002956 | 0.228 | A/T | ABCG2 |
| 4 | rs2622620 | 89063851 | 1325 | 5.048 | 1.702 | 8.395 | 0.003165 | 0.478 | A/C | ABCG2 |
| 7 | rs17327442 | 87212990 | 1325 | 6.631 | 2.209 | 11.05 | 0.003353 | 0.163 | A/T | ABCB1 |
| 7 | rs17327624 | 87216817 | 1325 | 6.111 | 2.018 | 10.2 | 0.003487 | 0.208 | T/G | ABCB1 |
| 7 | rs74491457 | 87348996 | 1325 | 6.77 | 2.1 | 11.44 | 0.004567 | 0.143 | G/A | RUNDC3B |
| 7 | rs28381724 | 87340535 | 1325 | 6.676 | 2.029 | 11.32 | 0.004937 | 0.142 | A/G | ABCB1 \| RUNDC3B |
| 7 | rs4148732 | 87234049 | 1325 | 6.6 | 1.974 | 11.23 | 0.005248 | 0.144 | C/T | ABCB1 |
| 7 | rs56227693 | 87200734 | 1325 | 17.93 | 5.277 | 30.58 | 0.005556 | 0.018 | C/G | ABCB1 |
| 7 | rs28746490 | 87297189 | 1325 | -16.05 | -27.45 | -4.655 | 0.005853 | 0.021 | T/C | ABCB1 \| RUNDC3B |
| 4 | 4:89066715 | 89066715 | 1325 | -4.609 | -7.929 | -1.288 | 0.006604 | 0.462 | C/A | ABCG2 |
| 7 | rs28381834 | 87200332 | 1325 | 17.27 | 4.746 | 29.79 | 0.006964 | 0.018 | T/G | ABCB1 |
| 7 | rs7787082 | 87157051 | 1325 | -6.266 | -10.83 | -1.7 | 0.007239 | 0.161 | A/G | ABCB1 |
| 7 | 7:87172053 | 87172053 | 1325 | -9.635 | -16.66 | -2.611 | 0.00727 | 0.05 | A/G | ABCB1 |
| 7 | rs10248420 | 87164986 | 1325 | -6.252 | -10.83 | -1.671 | 0.007566 | 0.161 | G/A | ABCB1 |
| 7 | rs181465270 | 87351586 | 1325 | 18.26 | 4.763 | 31.76 | 0.008111 | 0.015 | C/T | RUNDC3B |
| 4 | rs2725261 | 89036353 | 1325 | 4.562 | 1.189 | 7.935 | 0.008127 | 0.424 | T/C | ABCG2 |
| 7 | rs118078935 | 87189473 | 1325 | 17.24 | 4.447 | 30.03 | 0.00836 | 0.017 | C/T | ABCB1 |
| 4 | rs17731799 | 89068455 | 1325 | 4.524 | 1.166 | 7.881 | 0.008375 | 0.469 | T/G | ABCG2 |
| 7 | rs2373589 | 87151658 | 1325 | -6.16 | -10.74 | -1.578 | 0.008514 | 0.158 | T/C | ABCB1 |
| 4 | rs2622627 | 89065353 | 1325 | -4.458 | -7.782 | -1.133 | 0.008688 | 0.463 | C/A | ABCG2 |
| 4 | rs2725249 | 89065868 | 1325 | -4.458 | -7.782 | -1.133 | 0.008688 | 0.463 | A/C | ABCG2 |
| 4 | rs2725246 | 89068498 | 1325 | 4.497 | 1.137 | 7.858 | 0.008821 | 0.47 | A/G | ABCG2 |
| 4 | rs2622624 | 89069406 | 1325 | 4.485 | 1.118 | 7.852 | 0.009132 | 0.471 | C/T | ABCG2 |
| 4 | rs2725245 | 89068738 | 1325 | 4.474 | 1.112 | 7.837 | 0.009215 | 0.47 | A/G | ABCG2 |
| 7 | 7:87137015 | 87137015 | 1325 | 4.924 | 1.215 | 8.633 | 0.00937 | 0.262 | T/C | ABCB1 |
| 7 | rs6979885 | 87137461 | 1325 | 4.924 | 1.215 | 8.633 | 0.00937 | 0.262 | A/G | ABCB1 |
| 7 | rs56126905 | 87157049 | 1325 | 17.06 | 4.113 | 30 | 0.009908 | 0.017 | C/T | ABCB1 |
| 4 | rs2725247 | 89068257 | 1325 | 4.435 | 1.062 | 7.807 | 0.01007 | 0.472 | A/G | ABCG2 |
| 4 | rs34633905 | 89074771 | 1325 | 4.43 | 1.038 | 7.821 | 0.01058 | 0.471 | G/C | ABCG2 |
| 4 | rs2725242 | 89069527 | 1325 | -4.337 | -7.657 | -1.016 | 0.01059 | 0.463 | T/A | ABCG2 |
| 7 | rs150515188 | 87346068 | 1325 | -14.62 | -25.84 | -3.4 | 0.01076 | 0.022 | T/C | RUNDC3B |
| 7 | rs28401781 | 87148328 | 1325 | -7.401 | -13.09 | -1.712 | 0.01089 | 0.098 | T/C | ABCB1 |

CHR = chromosome; SNP = single nucleotide polymorphism; BP = base pair; L95 = lower 95% confidence interval; U95 = upper 95% confidence interval; MAF = minor allele frequency; GTPS = Guanosine-5′-triphosphates.

### **Supplementary Table 11.** Table of clinical outcomes by levels of rs2231142

| **Outcome** | **rs2231142** | **N** | **N Events** | **Median follow-up (years)** | **Max follow-up (years)** | **Patient years** | **Incidence rate (mean with 95% CI)*** |
| --- | --- | --- | --- | --- | --- | --- | --- |
| **Major bleeding** | G/G | 2214 | 78 | 1.81 | 3.93 | 4062.09 | 1.92 (1.52 - 2.40) |
|  | T/G | 549 | 24 | 1.88 | 3.81 | 996.75 | 2.41 (1.54 - 3.58) |
|  | T/T | 36 | 3 | 1.89 | 3.42 | 64.76 | 4.63 (0.96 - 13.54) |
| **Major or CRNM bleeding** | G/G | 2214 | 169 | 1.77 | 3.93 | 3981.39 | 4.24 (3.63 - 4.94) |
|  | T/G | 549 | 42 | 1.83 | 3.81 | 979.39 | 4.29 (3.09 - 5.80) |
|  | T/T | 36 | 3 | 1.89 | 3.42 | 64.76 | 4.63 (0.96 - 13.54) |
| **Stroke / Systemic embolism** | G/G | 2215 | 42 | 1.88 | 4.04 | 4345.57 | 0.97 (0.70 - 1.31) |
|  | T/G | 549 | 10 | 1.91 | 3.79 | 1062.1 | 0.94 (0.45 - 1.73) |
|  | T/T | 36 | 2 | 1.91 | 3.37 | 70.6 | 2.83 (0.34 - 10.23) |
| **Haemorrhagic stroke** | G/G | 2215 | 8 | 1.89 | 4.04 | 4368.91 | 0.18 (0.08 - 0.36) |
|  | T/G | 549 | 2 | 1.91 | 3.79 | 1071.38 | 0.19 (0.02 - 0.67) |
|  | T/T | 36 | 1 | 1.91 | 3.37 | 71.59 | 1.40 (0.04 - 7.78) |
| **Ischemic stroke** | G/G | 2215 | 23 | 1.88 | 4.04 | 4356.68 | 0.53 (0.33 - 0.79) |
|  | T/G | 549 | 8 | 1.91 | 3.79 | 1062.44 | 0.75 (0.33 - 1.48) |
|  | T/T | 36 | 1 | 1.91 | 3.37 | 70.63 | 1.42 (0.04 - 7.89) |

* Incidence rate calculated per 100 patient years.

CI = confidence interval; CRNM = clinically relevant non-major.

**References**

1. National Center for Biotechnology Information NIH National Library of Medicine 6624. 2022. <https://www.ncbi.nlm.nih.gov/gene/6624>. Accessed 2022-07-18.

2. Ward LD, Kellis M. HaploReg: a resource for exploring chromatin states, conservation, and regulatory motif alterations within sets of genetically linked variants. Nucleic Acids Res. 2012;40(Database issue):D930-4. doi:10.1093/nar/gkr917.

3. National Center for Biotechnology Information NIH National Library of Medicine 340578. 2022. <https://www.ncbi.nlm.nih.gov/gene/340578>. Accessed 2022-07-18.

4. National Center for Biotechnology Information NIH National Library of Medicine 869. 2022. <https://www.ncbi.nlm.nih.gov/gene/869>. Accessed 2022-07-18.

5. National Center for Biotechnology Information NIH National Library of Medicine 10716. 2022. <https://www.ncbi.nlm.nih.gov/gene/10716>. Accessed 2022-07-18.

6. National Center for Biotechnology Information NIH National Library of Medicine 1607. 2022. <https://www.ncbi.nlm.nih.gov/gene/1607>. Accessed 2022-07-18.

7. National Center for Biotechnology Information NIH National Library of Medicine 137868. 2022. <https://www.ncbi.nlm.nih.gov/gene/137868>. Accessed 2022-07-18.

8. Kwong A, Boughton AP, Wang M, VandeHaar P, Boehnke M, Abecasis G et al. FIVEx: an interactive eQTL browser across public datasets. Bioinformatics. 2021. doi:10.1093/bioinformatics/btab614.

9. Kanuri SH, Kreutz RP. Pharmacogenomics of Novel Direct Oral Anticoagulants: Newly Identified Genes and Genetic Variants. J Pers Med. 2019;9(1). doi:10.3390/jpm9010007.

10. Ueshima S, Hira D, Kimura Y, Fujii R, Tomitsuka C, Yamane T et al. Population pharmacokinetics and pharmacogenomics of apixaban in Japanese adult patients with atrial fibrillation. Br J Clin Pharmacol. 2018;84(6):1301-12. doi:10.1111/bcp.13561.

11. Lahteenmaki J, Vuorinen AL, Pajula J, Harno K, Lehto M, Niemi M et al. Pharmacogenetics of Bleeding and Thromboembolic Events in Direct Oral Anticoagulant Users. Clin Pharmacol Ther. 2021;110(3):768-76. doi:10.1002/cpt.2316.

12. Ueshima S, Hira D, Fujii R, Kimura Y, Tomitsuka C, Yamane T et al. Impact of ABCB1, ABCG2, and CYP3A5 polymorphisms on plasma trough concentrations of apixaban in Japanese patients with atrial fibrillation. Pharmacogenetics and genomics. 2017;27(9):329-36. doi:10.1097/FPC.0000000000000294.

13. Eliquis European Public Assessment Report. <https://www.ema.europa.eu/en/medicines/human/EPAR/eliquis>.
